# 3D-printed nerve guidance conduits multi-functionalized with canine multipotent mesenchymal stromal cells promote neuroregeneration after sciatic nerve injury in rats

**DOI:** 10.1186/s13287-021-02315-8

**Published:** 2021-05-29

**Authors:** Diego Noé Rodríguez-Sánchez, Giovana Boff Araujo Pinto, Luciana Politti Cartarozzi, Alexandre Leite Rodrigues de Oliveira, Ana Livia Carvalho Bovolato, Marcio de Carvalho, Jorge Vicente Lopes da Silva, Janaina de Andréa Dernowsek, Marjorie Golim, Benedito Barraviera, Rui Seabra Ferreira, Elenice Deffune, Mathues Bertanha, Rogério Martins Amorim

**Affiliations:** 1 Department of Veterinary Clinics, School of Veterinary Medicine and Animal Science, São Paulo State University (UNESP), Botucatu, SP Brazil; 2 Department of Structural and Functional Biology, Institute of Biology, University of Campinas, Campinas, SP Brazil; 3 Blood Transfusion Center, Cell Engineering Laboratory, Botucatu Medical School, São Paulo State University, Botucatu, SP Brazil; 4 Renato Archer Information Technology Center (CTI), Three-dimensional Technologies Research Group, Campinas, SP Brazil; 5 Hemocenter division of Botucatu Medical School, São Paulo State University, Botucatu, SP Brazil; 6 Center for the Study of Venoms and Venomous Animals (CEVAP), São Paulo State University (UNESP), Botucatu, SP Brazil

**Keywords:** Canine mesenchymal stem cells, Nerve regeneration, Sciatic nerve injury, Cell-based therapy, Tissue engineering, Nerve guidance conduits, 3D printing, Fibrin, Scaffold

## Abstract

**Background:**

Nerve injuries are debilitating, leading to long-term motor deficits. Remyelination and axonal growth are supported and enhanced by growth factor and cytokines. Combination of nerve guidance conduits (NGCs) with adipose-tissue-derived multipotent mesenchymal stromal cells (AdMSCs) has been performing promising strategy for nerve regeneration.

**Methods:**

3D-printed polycaprolactone (PCL)-NGCs were fabricated. Wistar rats subjected to critical sciatic nerve damage (12-mm gap) were divided into sham, autograft, PCL (empty NGC), and PCL + MSCs (NGC multi-functionalized with 10^6^ canine AdMSCs embedded in heterologous fibrin biopolymer) groups. In vitro*,* the cells were characterized and directly stimulated with interferon-gamma to evaluate their neuroregeneration potential. In vivo*,* the sciatic and tibial functional indices were evaluated for 12 weeks. Gait analysis and nerve conduction velocity were analyzed after 8 and 12 weeks. Morphometric analysis was performed after 8 and 12 weeks following lesion development. Real-time PCR was performed to evaluate the neurotrophic factors BDNF, GDNF, and HGF, and the cytokine and IL-10. Immunohistochemical analysis for the p75^NTR^ neurotrophic receptor, S100, and neurofilament was performed with the sciatic nerve.

**Results:**

The inflammatory environment in vitro have increased the expression of neurotrophins BDNF, GDNF, HGF, and IL-10 in canine AdMSCs. Nerve guidance conduits multi-functionalized with canine AdMSCs embedded in HFB improved functional motor and electrophysiological recovery compared with PCL group after 12 weeks. However, the results were not significantly different than those obtained using autografts. These findings were associated with a shift in the regeneration process towards the formation of myelinated fibers. Increased immunostaining of BDNF, GDNF, and growth factor receptor p75^NTR^ was associated with the upregulation of BDNF, GDNF, and HGF in the spinal cord of the PCL + MSCs group. A trend demonstrating higher reactivity of Schwann cells and axonal branching in the sciatic nerve was observed, and canine AdMSCs were engrafted at 30 days following repair.

**Conclusions:**

3D-printed NGCs multi-functionalized with canine AdMSCs embedded in heterologous fibrin biopolymer as cell scaffold exerted neuroregenerative effects. Our multimodal approach supports the trophic microenvironment, resulting in a pro-regenerative state after critical sciatic nerve injury in rats.

## Background

Peripheral nervous system (PNS) injuries are debilitating and result in long-term sensorimotor defects, leading to a negative quality of life in dogs and humans beings [1, 2]. In dogs, injuries of the brachial plexus or sciatic nerve are common [2–4]. In humans, epidemiological studies showed a PNS injury incidence rate of 13.9 individuals per 100,000 habitants per year and 2–5% of patients admitted to level I trauma centers might have PNS injuries [5]. In this context, peripheral nerve injuries occurring naturally in dogs display similar features with human disease hold promise for providing predictive proof of concept in the evaluation of new therapeutics and bioengineering devices [6].

Complete regeneration of nerves does not occur in critical lesions with long gaps [7, 8]. The distal stump in lesions with long-gap defects does not respond to trophic signals released by the proximal stump, resulting in poor nerve regrowth [9]. This regenerative response is associated with a complex interaction between the Wallerian degeneration process, the immunological response, Schwann cells, and pro-regenerative molecules such as neurotrophic factors and cytokines [9]. Autografting is the current standard treatment for nerve injuries, resulting in long-gap defects [10]. However, this procedure has several disadvantages, such as additional damage to donor nerves, and insufficient revascularization [10]. These limitations have led to the development of nerve guidance conduits (NGCs) for nerve repair that guide regenerating axons, support vascularization, increase the concentration of trophic factors, and avoid the formation of scarred tissue [11, 12].

Types of NGCs, namely, synthetic (e.g., polyglycolic acid [PGA] and polycaprolactone [PCL]) and biological (e.g., veins, arteries, or collagen), have been studied as alternatives to autografts in short-gap models. However, the functional results of such conduits were not superior to those of autografts in long-gap defects [10, 13–17]. Typically, conventional fabrication techniques can only result in the development of NGCs with simple architectures (e.g., straight hollow conduits) with limited choices of materials and dimensions [18]. Inferior results obtained using hollow NGCs were associated with insufficient migration of Schwann cells and lack of pro-regenerative molecules [19].

Additive manufacturing, also known as three-dimensional (3D) printing, is a process of joining materials to make parts from 3D model data, usually layer upon layer, as opposed to subtractive and formative manufacturing methodologies [18, 20, 21]. Therefore, this robotics-based biomanufacturing approach can be used for the development of biocompatible tissue repair constructs with high flexibility and geometric freedom offering a differential advantage for medical devices production [18, 20, 21], 3D printing offers the possibility to control the architecture using biocompatible polymers [18, 22, 23]. NGCs manufactured via 3D printing vary in complexity and size [21–23]. The advantages of 3D printing constructs include mechanical stability, pore interconnectivity, and customizability [18, 23]. Polycaprolactone (PCL) is a thermoplastic, non-toxic, biodegradable, and hydrophobic polymer widely used as a scaffold biomaterial in vivo and can be adapted to 3D printing [11, 24–27]. Nerve guidance conduits (NGCs) constructed with PCL proved to be an adequate substrate for the survival and differentiation of Schwann cells and mesenchymal stromal cells (MSCs) [27, 28].

The combination of NGCs, extracellular matrix, cells, and growth factors and their interactions could be potential tools for restoring damaged nerve tissue [11, 12]. The efficiency of Schwann cells has been demonstrated; however, certain limitations are associated, including isolation and expansion under ex vivo conditions [29]. Due to their proliferative ability and easy accessibility, adipose-tissue-derived MSCs (AdMSCs) exhibit a translational potential [30–33]. Previous studies have demonstrated the potential of MSCs to secrete powerful neurotrophic factors as well as anti-inflammatory and immunomodulatory molecules, thereby favoring nerve regeneration [30, 34–36].

The paracrine activity of AdMSCs is dependent on their viability and homing into the local inflammatory microenvironment; however, direct injection leads to poor engraftment and leakage of the cells [31, 37]. In this study, we used a scaffold composed of heterologous fibrin biopolymer (HFB) derived from snake venom (i.e., *Crotalus durissus terrificus*) that is non-cytotoxic, biodegradable with adhesive and sealant properties to retain the AdMSCs into the internal wall of the NGC [38–41]. In vivo, the HFB provided adequate adhesion of rootlets after lesioning in rodents [42–46]. We hypothesized that the multi-functionalization of PCL-NGCs manufactured by 3D printing with canine AdMSCs embedded in fibrin biopolymer can enhance nerve regeneration following the repair of critical nerve injury in rats.

## Methods

### Experimental design

Female Wistar rats (*Rattus novergicus*) with weights in the range of 200–300 g were used for the experimental procedures. The rats were maintained under controlled humidity, temperature, and constant light/dark cycles. All procedures were performed in accordance with the ethical principles set forth by the National Council of Animal Experimentation (CONCEA) and with the approval of the Ethics Committee in Animal Experimentation of São Paulo State University (CEUA/FMB, UNESP, protocol no. 1243–2017). The animals were divided into four groups. In the sham group (*n* = 5), the sciatic nerve was surgically exposed without any changes. The proximal and distal segments were resected, forming a gap of 12 mm, and sutured with perineural stitches in the animals of the autograft group (*n* = 5). In the PCL group, a gap of 12 mm was formed with nerve resection, and an NGC empty was fixed (*n* = 5). In the PCL + MSCs group, a gap of 12 mm was formed, and the NGC was fixed and multi-functionalized with AdMSCs embedded in HFB (*n* = 5). The sciatic functional index (SFI) and tibial functional index (TFI) were evaluated in vivo for 12 weeks after injury. Gait analysis was evaluated using the Catwalk system, and nerve conduction velocity (NCV) was measured at 8 and 12 weeks. Morphometric analysis was performed 8 and 12 weeks post-injury. To evaluate the production of neurotrophic factors at 4 weeks, brain-derived neurotrophic factor (BDNF), glial cell line-derived neurotrophic factor, hepatocyte growth factor (HGF), and the cytokine and interleukin-10 (IL-10) in the spinal cord, real-time PCR (RT-qPCR) was performed nerve in both sham and PCL + MSCs groups (*n* = 3). In addition, immunohistochemical analysis of the sciatic nerve for BDNF, GDNF, p75 neurotrophin receptor (p75^NTR^), S-100, and neurofilament were performed in both sham and PCL + MSCs groups (*n* = 3).

### Isolation, differentiation, and characterization of canine AdMSCs

Subcutaneous canine adipose tissue was obtained from healthy young female dogs undergoing elective surgery in accordance with a previously published protocol [47]. Adipose tissue was digested in 0.04% type 1A collagenase (1 mg/mL, Thermo Fisher Scientific, São Paulo, Brazil) for 1 h at 37 °C with gentle shaking. Digested tissue was blocked, centrifuged, and filtered (BD Falcon cell strainer, 70 μm, San Jose, CA, USA). Canine AdMSCs were isolated based on their inherent property of plastic adherence in culture media containing 90% Dulbecco’s modified Eagle’s medium (DMEM), 10% fetal bovine serum (FBS), and 1% penicillin/streptomycin (100 U/mL) (all from Gibco, Grand Island, NY, USA). Cellular expansion was continued until the third passage, and the cells were cryopreserved to induce differentiation, for immunophenotypic analysis, and transplantation later on.

Canine AdMSCs were tested for their ability to differentiate into adipocyte, osteoblast, and chondrocyte lineages. Differentiation was induced in cells that underwent third passage using StemPro adipogenesis, chondrogenesis, and osteogenesis differentiation kits (Gibco, Grand Island, NY, USA) following the manufacturer’s recommendations. The cells were fixed in paraformaldehyde (4%, pH 7.34) 2 weeks after stimulation, and the evaluation of osteogenic and adipogenic differentiation were performed using histological stains, namely, Alizarin Red (2%, pH 4.2) and Oil Red (0.5% in isopropanol) (Sigma-Aldrich, Saint Louis, MA, USA), respectively. Three weeks after chondrogenic differentiation, the cells cultured as a micromass were fixed in 10% formalin, embedded in paraffin, and stained with hematoxylin-eosin. Samples were analyzed and photographed under an inverted light microscope using LAS 4.0 software (DM IRB; Leica Microsystems, Wetzlar, Germany).

Canine AdMSCs were characterized by the presence of the surface marker CD90 or absence of surface markers CD45, CD34, and CD71 [48, 49]. The concentration of cells in the third passage was counted and adjusted to 1 × 10^5^ cells. Subsequently, the cells were incubated with primary antibody conjugates CD90-PerCP (BD Pharmigen™, San Diego, CA, USA), CD71-FITC (BD Pharmigen™), CD45-PE (BD Pharmigen™), and CD34-FITC (BD Pharmigen™). Antibodies were incubated for 30 min at room temperature. Cells were then washed with phosphate-buffered saline (PBS), and FACSCalibur® 4-color cytometer (Becton Dickinson Company, San Jose, CA, USA) was used to acquire and analyze the samples, standardizing a total of 2 × 10^4^ events collected per tube. Cells incubated without primary antibodies were used as controls to distinguish non-specific fluorescence. The gate on canine AdMSC population was based on the parameters of size (forward scatter) versus cell granularity (side scatter), following the phenotypic characterization. The analyses were performed using CellQuestPro® and FlowJo® software.

### Stimulation of canine AdMSCs with interferon-gamma

Canine AdMSCs were activated via direct stimulation to evaluate the properties of neurotrophic and anti-inflammatory molecules using a recombinant inflammatory mediator relevant to nerve injury, following a previously described protocol with minor modifications [50]. Cells were stimulated with canine interferon-gamma (IFN-γ) in the third passage. Triplicates were obtained with 2 × 10^5^ cells/cm^2^ per well in a 24-well plate (Costar®, TC-treated, Corning, NY, USA). Subsequently, cells were stimulated with 0.75 mL basal medium containing IFN-γ (50 ng/mL, IFN- γ canine recombinant; Kingfisher Biotech, Saint Paul, USA) for 96 h. At this point, the cells were collected using TRIzol reagent (Invitrogen, São Paulo, Brazil) and stored at − 80 °C for RNA extraction and analysis of gene expression. For the control, cells were cultured in basal culture medium containing DMEM and 10% FBS (all from Gibco).

Gene expression of neurotrophic factors (BDNF, GDNF, and HGF) and anti-inflammatory molecules (IL-10) was quantified. Cells were lysed and homogenized with TRIzol reagent, and RNA extraction was performed using the Mini RNAeasy kit (Qiagen, São Paulo, Brazil). RNA was eluted with RNA-free water and quantified and analyzed by spectrophotometry using a NanoDrop 2000 spectrophotometer (Thermo Fisher Scientific, Wilmington, USA) for the absorbance ratios 260/280 nm and 260/230 nm. Total RNA extracted from the cells was of high quality and purity, indicating that the extraction method was efficient. cDNA was synthesized using a High-Capacity cDNA Reverse Transcription Kit (Applied Biosystems™, Life Technologies Corporation, Carlsbad, USA), followed by amplification using a Veriti 96 Well Thermal Cycler (Life Technologies, Carlsbad, USA). The cDNA samples were cryopreserved and used as templates for PCR reactions.

The reactions were performed in triplicate, using the cDNA produced in previous steps as a template, with a PowerUp SYBR Green Master Mix (Applied Biosystems™, Life Technologies, Carlsbad, CA, USA), RNA-free water, and canine primers (Thermo Fisher Scientific, São Paulo, Brazil) (Additional file 1: Table S1). The samples were tested with two reference genes, glyceraldehyde-3-phosphate dehydrogenase (GAPDH) and hypoxanthine phosphoribosyltransferase (HPRT). The qPCR reaction was performed using the QuantStudio™ 12 K Flex Real-Time PCR System thermocycler (Life Technologies, Carlsbad, USA) with the following parameters: 50 °C for 2 min, 95 °C for 2 min, and 45 cycles of 95 °C for 1 s and 60 °C for 30 min. Relative quantification of expression of the genes of interest was performed using the ΔΔCt method [51].

### Fabrication and assembly of NGCs

The NGCs were assembled from 3D-printed PCL membranes. The membrane fabrication was based on a material extrusion process called fused filament fabrication (FFF) using FAB@CTI (Renato Archer Information Technology Center - CTI, São Paulo, Brazil), an experimental 3D printing platform [52]. Previously, the filament extrusion head was adapted to different diameters and melting temperatures, which allowed the molding of a thermoplastic polymer via an orifice (open-ended die) [53]. Previous studies have evaluated the interactions between MSCs and 3D-printed PCL matrices [54]. The printing parameters were defined using FAB@CTI software (Renato Archer Information Technology Center - CTI, São Paulo, Brazil). The following parameters were set: jog speed 2400 Hz, deposition rate 0.07, path speed 8.8 mm/s, path width 0.3 mm, path height 0.3 mm, and temperature of 80 °C. The 3D-printed membranes were sputter-coated with gold (MED 010; Balterz Union) and visualized using a scanning electron microscope (ESEM Quanta 200; Fei Company, Oregon, USA). The geometric parameters were evaluated using image analysis software (ImageJ, National Institute of Health, Bethesda, USA). During the assembly of NGCs, the membranes were wrapped around a 1.5-mm support and sealed with controlled heating. The NGCs were sterilized by washing with a 70% ethanol solution for 10 s, followed by washing with distilled water. After drying at room temperature, the NGCs were subjected to UV irradiation (200–280 nm) for 2 h.

### Heterologous fibrin biopolymer (HFB) scaffold

The HFB was kindly supplied in sufficient quantity for this study by the Center for the Study of Venoms and Venomous Animals at São Paulo State University, Brazil. The components and formula of the applied HFB are contained in its patents (registry number: BR1020140114327 and BR1020140114360). The product is distributed in three vials, stored at − 20 °C, and must be mixed and applied immediately at the site of interest [39–44].

### Experimental injury and repair with NGCs

**Fig. 1:**
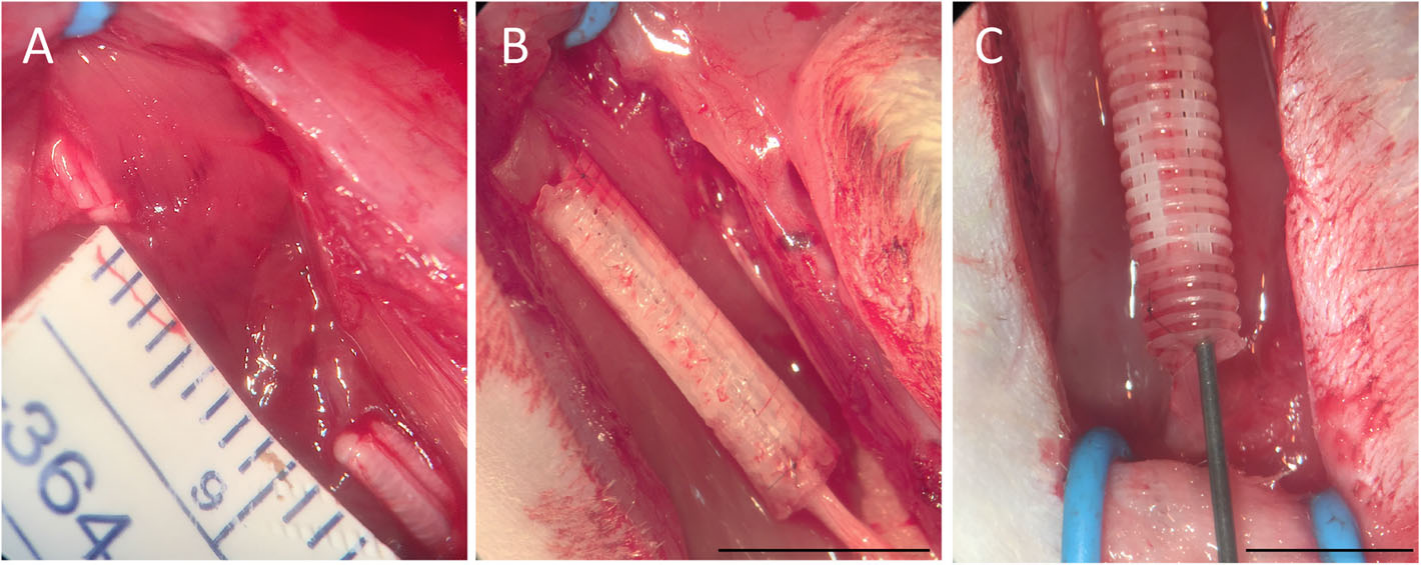
Experimental sciatic injury and repair in the autograft, PCL, and PCL + MSC groups. **a** The experimental lesion consisted of a gap of 12 mm, which was considered to be above the experimental critical level in rats. **b** Nerve stumps were introduced and fixed into the NGC in the PCL group. Scale bar: 1 cm. **c** NGCs were multi-functionalized with 1 × 10^6^ canine AdMSCs embedded in HFB. Scale bar: 0.5 cm

Sciatic nerve experimental injury was induced in rats under the influence of anesthesia containing isoflurane (Isoforine®; Cristalia, São Paulo, Brazil) using a microsurgical microscope (DF Vasconcelos, Rio de Janeiro, Brazil). The experimental lesion consisted of a gap of 12 mm, which was considered to be above the experimental critical level in rats [55] (Fig. 1a). In the sham group, the nerves were exposed without any modifications. In the autograft group, the proximal and distal segments were resected, inducing a gap of 12 mm and suturing with perineural stitches (9/0 Ethilon, Ethicon, Cincinnati, USA). In the PCL group, nerve stumps were introduced and fixed 1 mm into the NGC (9/0 Ethilon, Ethicon, Cincinnati, USA) (Fig. 1b). In the PCL + MSC group, nerve stumps were introduced and fixed 1 mm into the NGC (9/0 Ethilon, Ethicon, Cincinnati, USA).

Thereafter, NGCs were multi-functionalized with 1 × 10^6^ canine AdMSCs embedded in HFB that has been previously tested as a cell scaffold [40, 41, 56]. Fibrin polymerizes rapidly following the mixing of three components, namely, cryoprecipitated from water buffalo (*Bubalus bubalis*) blood, calcium chloride, and thrombin-like protein purified from South American rattlesnake (*Crotalus durissus terrificus*) [39, 41]. First, 10^6^ AdMSCs were mixed with 25 μL of cryoprecipitated. The nerve guidance conduit was loaded slowly and homogeneously with a cryoprecipitated + AdMSC solution using a microsyringe (50 μL, 22 s-gauge, point style 2; Hamilton, Nevada, USA) (Fig. 1c). Subsequently, a solution of 12.5 μL of calcium chloride and 12.5 μL thrombin-like was administered, resulting in a final suspension with a volume of 50 μL. This process allowed the formation of a homogeneous cell/fibrinogen suspension into the NGC at the first step, which was coagulated after contact with thrombin + CaCl_2_ within the NGC. Following surgical procedures, the musculature was co-opted in layers. Rats were administered tramadol intraoperatively (20 mg/kg/SC) and in the postoperative periods (2.5 mg/day in water for 5 days).

### Sciatic and tibial nerve functional indices

Functional indices were evaluated preoperatively and weekly during the 12-week observation period in the sham, autograft, PCL, and PCL + MSC groups. The plantar surface of the hind limbs was moistened with black ink. The rats walked with a standard walk trace on a sheet of white paper where the footprints were recorded. Subsequently, the distance between the third toe and the hind limb pads (print length, PL), the first and the fifth toes (toe spread, TS), and the second and fourth toes (intermediary toe spread, ITS) were measured. These parameters were evaluated with the right (lesioned) and left (non lesioned) hind limbs, and the values were calculated using the following formulas described by Bain et al. [57]: sciatic functional index: − 38.3 ([EPL-NPL]/NPL) + 109.5 ([ETS-NTS]/NTS) + 13.3 ([EIT-NIT]/NIT) − 8.8 (30, 31); IFP = 174.9 (EPL˗NPL/NPL) + 80.3 (ETS˗NTS/NTS) − 13.4; tibial functional index: − 37.2 ([EPL-NPL]/NPL) + 104.4 ([ETS-NTS]/NTS) + 45.6 ([EIT-NIT]/NIT) − 8.8. Sciatic and tibial functional indices equal to − 100 indicated total impairment of the sciatic and posterior tibial nerves, whereas values oscillating around 0 reflected a normal function of the three nerves. The mean ± standard deviation was calculated with three gait cycles for each experimental group each week.

### Gait analysis

Functional locomotor recovery was evaluated using the CatWalk System (Noldus, Wageningen, Netherlands). Catwalk analysis was performed preoperatively and after 8 and 12 weeks in sham, autograft, PCL, and PCL + MSC groups. The CatWalk walkway consisted of a glass roof (100 × 15 × 0.6 cm). Rats were placed on the CatWalk walkway and allowed to walk freely. The LED light emitted from an encased fluorescent lamp was reflected along the glass plate, thereby intensifying the areas on which the front limbs and hind limbs were in contact with the glass plate. The contact areas were captured by a high-speed video camera positioned underneath the glass plate connected to a computer running Catwalk software v10.5 (Noldus). The camera was calibrated, and the signals were digitized, frame-by-frame, using the PCImage-SG video card (Matrix vision GmH, Oppenheimer, Germany) and sent to the matrix for classification. Three runs were performed and classified from each animal and the parameters were obtained for each animal at each time point. The following parameters were recorded: maximum contact area (ipsilateral (left)/contralateral (right) ratio), maximum contact intensity (ipsilateral (left)/contralateral (right) ratio), swing speed (ipsilateral (left)/contralateral (right) ratio), and swing (seconds) (swing exercised by the limbs when they are not in contact with the glass plate) and stand time (seconds).

### Nerve conduction velocity

Nerve conduction velocity (NCV) was calculated preoperatively and after 8 and 12 weeks in the sham, autograft, PCL, and PCL + MSC groups, according to a previously published protocol [58, 59]. Under anesthesia, the sciatic nerve was stimulated with single electrical pulses (200-μs duration) and supramaximal stimulation that ensured maximal amplitude. Using needle electrodes, the sciatic nerve was percutaneously stimulated proximal to the lesion site at the level of the sciatic notch and distal to the lesion at the level of the ankle. Compound muscle action potentials (cMAP) of the plantar muscles were recorded using monopolar needles inserted into the muscle bellies and displayed with an oscilloscope (Sapphire II 4ME; Teca medelec, USA). Motor NCV was calculated by dividing the distance between stimulation sites by the average latency evoked from two sites (sciatic notch and ankle). The mean ± standard deviation was calculated for each experimental group and at each evaluated time point.

### Specimen preparation and morphometric analysis

Nerves were harvested after 8 and 12 weeks from the sham, autograft, PCL, and PCL + MSC groups. Under general anesthesia with isoflurane (Isoforine®; Cristalia, Brazil), rats were euthanized with barbiturate overdose (Thiopentax, Cristalia, São Paulo, Brazil). The vascular system was rinsed by transcardial perfusion with phosphate-buffered saline (PBS; 0.1 M, pH 7.4). Fixation was performed in 2% glutaraldehyde and 1% paraformaldehyde in PBS (0.2 M, pH 7.34), and nerves containing NGC were immersed in the same solution for 24 h at 4 °C. The sciatic nerve segment into the NGC was dissected and divided into two parts: proximal and distal. Nerves were washed with PBS (0.1 M, pH 7.4) and post-fixed for 3 h in 1% osmium tetroxide solution mixed with PBS (pH 7.4). The specimens were dehydrated and embedded in glycol methacrylate resin (Leica Microsystems, Heidelberg, Germany). The blocks were trimmed, and semi-thin sections (1–2 μm) were obtained with an ultramicrotome (Leica RM 2265; Leica Microsystems CMS), which were stained with toluidine blue (0.25%). Morphometric analysis was performed by sampling at least 30% of the cross section of each nerve using a bright-field microscope (Leica DM 4000 B-M; Leica Microsystems CMS) [60]. The analysis was performed with two sampled fields from each nerve (magnification of × 100) using Adobe Photoshop CC 2019. Morphometric parameters evaluated included myelinated axon diameter, myelinated fiber diameter, myelin thickness (fiber diameter − axon diameter/2), and “g” ratio (axon diameter/fiber diameter). The mean ± standard deviation was calculated for each experimental group and at each evaluated time point.

### Immunohistochemical study of sciatic nerve and RT-qPCR analyses of spinal cord samples

Immunohistochemical analysis (S-100, neurofilament, BDNF, GDNF, and p75^NTR^) of sciatic nerve samples and qPCR of the spinal cord samples (BDNF, GDNF, HGF, and IL-10) were performed for the sham and PCL + MSC (*n* = 3) groups after 4 weeks. Rats were euthanized with barbiturate overdose (Thiopentax; Cristália). The vascular system was rinsed by transcardial perfusion with phosphate-buffered saline (PBS; 0.1 M, pH 7.4). Using the nippers, the dorsal side of the spinal column was gently opened proceeding in a cranial to caudal direction by making one or two snips on either side and clipping the resulting flap of bone free. Then, the spinal cord was slowly eased out using microscissors. Fresh lumbar segments (L3–S1) at the T13–L1 vertebral level were harvested. Using a microsurgical microscope, ventral fissure of spinal cord was identified and sectioned to obtain the tissue ipsilateral to the lesion. The specimen was frozen in liquid nitrogen and stored at − 80 °C.

After spinal cord harvesting, carcass were fixed in 4% paraformaldehyde in PBS (0.1 M, pH 7.34), and the regenerated nerve was dissected and immersed in the same solution for 12 h at 4 °C. Specimens were immersed in ascending order 10%, 20%, and 30% of the sucrose solutions (0.1 M PBS, pH 7.4) for 12 h, mixed with Tissue-Tek OCT (Sakura Finetek, Torrance, USA), and frozen at − 80 °C.

Longitudinal cryostat sections (12 μm) of the sciatic nerves were acclimatized, washed, and incubated in 3% bovine serum albumin solution or 3% donkey serum in PBS (0.1 M, pH 7.4) for 1 h, followed by incubation in a moist chamber with primary antibodies against S100, neurofilament H (NF), BDNF, GDNF, and p75^NTR^ for 4 h (Additional file 2: Table S2). After rinsing with PBS, the sections were incubated with Alexa Fluor 488, Alexa Fluor 546, or CY2-conjugated secondary antiserum for 45 min at room temperature. The sections were then mounted in a mixture of glycerol/PBS (3:1) for quantitative measurements or glycerol/DAPI for qualitative analysis. Representative images were obtained using a fluorescence microscope (BX51; Olympus Corporation, Tokyo, Japan) equipped with a camera (DP 72; Olympus Corporation). Four images of each sample were imported for the determination of the integrated pixel density that represented the intensity of labeling using ImageJ software (version 1.33u, National Institutes of Health, USA), according to a previously published protocol [61, 62]. The mean intensity ± standard deviation was calculated for each group.

For RT-qPCR, the spinal cord was finely pricked and homogenized in TRIzol reagent (TRIzol™, Invitrogen, São Paulo, Brazil) and chloroform. The samples were vigorously shaken for 30 s using a Precellys Lysing Kit® (Uniscience, São Paulo, Brazil) with a Precellys 24 tissue homogenizer (Bertin Technologies SAS, Montigny-le-Bretonneuz, France). Total RNA was extracted, quantified, and reverse-transcribed to cDNA, which was amplified as described previously in the RT-qPCR assay procedure performed with cells. Assays analyzing the levels of BDNF, GDNF, HGF, and IL-10, were performed (all from Thermo Fisher Scientific, São Paulo, Brazil) (Additional file 3: Table S3). Samples were tested with two reference genes, β2-microglobulin and HPRT. The qPCR reaction was performed using the QuantStudio™ 12 K Flex Real-Time PCR System thermocycler (Life Technologies System, Carlsbad, USA) with the following parameters: 50 °C for 2 min, 95 °C for 2 min, and 45 cycles of 95 °C for 1 s and 60 °C for 30 min. Relative quantification of expression of the genes of interest was performed using the ΔΔCt method [51].

### Statistical analysis

Variables, namely, sciatic and tibial functional indices, Catwalk analysis, and NCV were assessed for normality with statistical tests (Shapiro–Wilk or Kolmogorov–Smirnov), descriptive statistics, and graphic analyses (QQ plot). An analysis of variance test (two-way ANOVA, multiple comparisons) was performed followed by Tukey’s test to verify the differences in the means of the variables between each group and the time of the experiment. Other variables (integrated pixel density and relative quantification) were assessed for normality using statistical tests (Shapiro–Wilk), descriptive statistics, and graphic analysis. For parametric data, the *t*-test was performed with unpaired samples. For non-parametric data, the Mann-Whitney test was performed for unpaired samples. The level of significance between the groups was set at *p* < 0.05. The differences were denoted by a single asterisk (*p* < 0.05), two asterisks (*p* < 0.01), or three asterisks (*p* < 0.001) (GraphPad Prism version 8 for Mac, San Diego, USA).

## Results

### Canine AdMSCs showed mesenchymal fate and differentiation potential

Following isolation, AdMSCs demonstrated a homogeneous appearance and fusiform morphology during the first week after passage zero and reached 80% confluence and formed a monolayer in 2 weeks. Multipotentiality was detected in vitro via tri-lineage differentiation into adipocytes, osteoblasts, and chondrocytes. Alizarin red staining demonstrated the formation of an extracellular calcium matrix after 21 days. Oil Red staining confirmed the presence of intracytoplasmic lipid deposits after 14 days. Staining with toluidine blue demonstrated the deposition of the extracellular matrix after 21 days (Additional file 4: Figure S1). Immunophenotypic analysis of AdMSCs by flow cytometry confirmed the positive expression of CD 90 (Thy-1) and the absence of expression of hematopoietic and endothelial antigens CD45 and CD34 (transmembrane glycoproteins) and CD71 (transferrin receptor) (Additional file 4: Figure S1).

### Canine AdMSCs enhanced their trophic and anti-inflammatory potential after in vitro stimulation

**Fig. 2:**
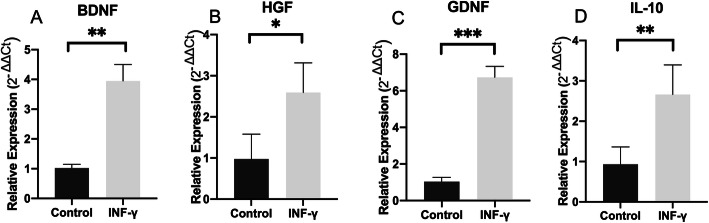
Relative gene expression after 96 h following AdMSC stimulation with interferon-gamma (IFN-γ). **a** Relative expression of BDNF, **b** GDNF, **c** HGF, and **d** IL-10. The samples were tested with two reference genes, glyceraldehyde-3-phosphate dehydrogenase (GAPDH) and hypoxanthine phosphoribosyl transferase (HPRT). Data are represented as mean ± SEM. *p* < 0.05*; *p* < 0.01**; *p* < 0.001***

Gene expression of neurotrophic factors BDNF, GDNF, and HGF in AdMSCs was higher following stimulation with IFN-γ (BDNF 3.94 ± 0.55, GDNF 6.7 ± 0.59, and HGF 2.5 ± 0.36) than in unstimulated AdMSCs (BDNF 1.02 ± 0.12, GDNF 1.03 ± 0.23, and HGF 0.97 ± 0.60) (BDNF *p* = 0.02; GDNF *p* < 0.001, and HGF *p* = 0.01). The expression of cytokine IL-10 was significantly higher in AdMSCs following stimulation with IFN-γ (2.66 ± 0.36) than in unstimulated AdMSCs (0.93 ± 0.21) (*p* = 0.07) (Fig. 2a–d).

### Ultrastructural analysis of 3D-printed NGCs

**Fig. 3:**
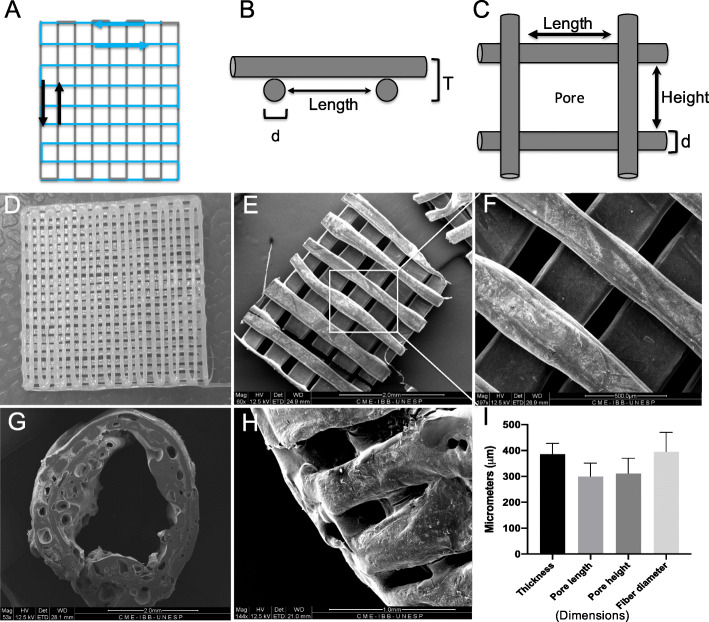
Geometric characterization of NGG-PCL 3D-printed membranes. **a** Height and length path of the first and second layers during 3D printing. **b** PCL membranes: lateral view showing two layers (T) and diameter (d) of the filaments with 396 ± 74 μm. **c** PCL membrane dorsal view showing the pores with 312 ± 58 μm of length and 300 ± 51 μm of height. **d** 3D-printed PCL membrane macroscopic view. **e** Internal face of PCL membrane (scale bar: 2.0 mm). **f** Scanning microscopy electronic images of 3D-printed PCL membranes with different pores, filament lengths, and deposition of two layers (scale bar: 500 μm). **g** NGC cross sectional view after assembly (scale bar: 2.0 mm). **h** NGC external surface view (scale bar 2.0 mm). **i** Measures of geometric parameters from PCL membranes (*n* = 5). Values are represented as mean ± SEM

The NGCs were manufactured by 3D printing with PCL membranes using the FFF technique. PCL filaments (diameter of 396 ± 74 μm) were continuously deposited in a square geometry along the vertical direction for the first layer and the lateral direction for the second layer, resulting in the formation of a bilayer membrane with a thickness of 386 ± 41 μm, and an area of 225 mm^2^ (Fig. 3a–d). The air gaps between the filaments (areas without polymer) formed pores with a height of 312 ± 58 μm and a length of 300 ± 51 μm, as depicted in Fig. 3e–i. The membranes were rolled and sealed with controlled heating (Fig. 3g). Smooth architecture was observed on the outer surface (Fig. 3h).

### NGCs multi-functionalized showed positive functional motor recovery

**Fig. 4:**
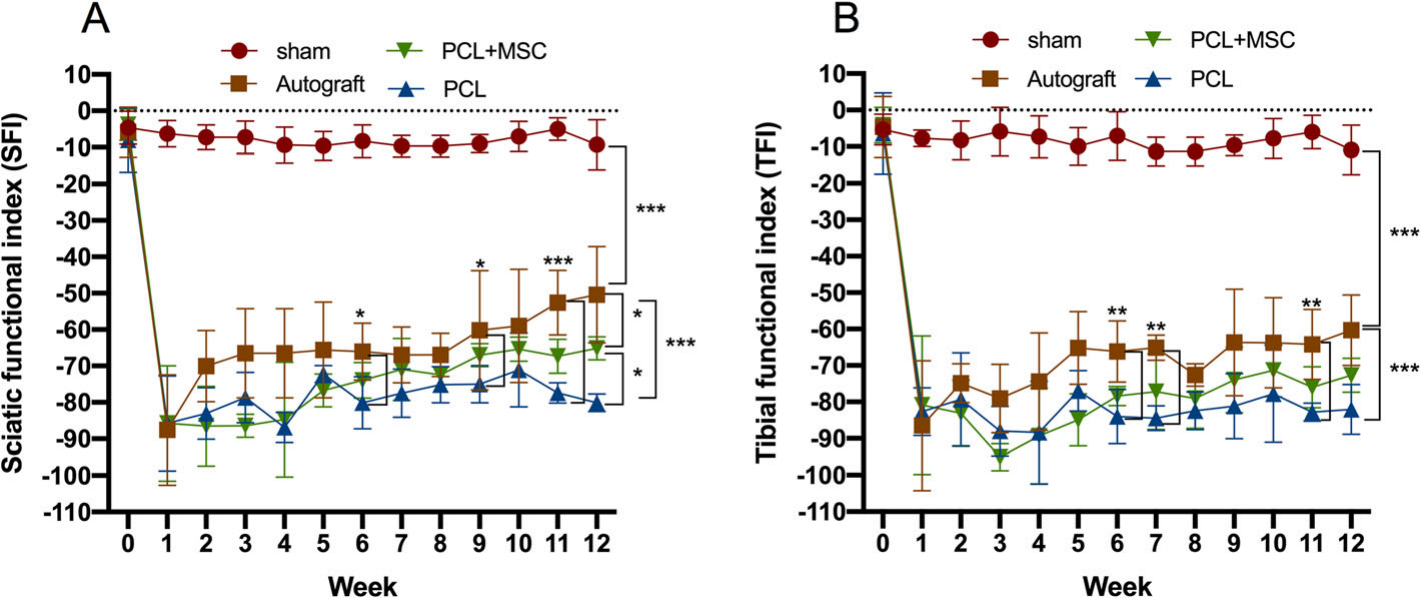
Sciatic nerve functional index (SFI) and tibial functionality index (TFI) during 12 weeks in the Sham, autograft, PCL, and PCL + MSC groups. **a** SFI. **b** TFI. The values we obtained weekly and are represented as mean ± SEM. *p* < 0.05*; *p* < 0.01**; *p* < 0.001***

After 9 weeks, SFI analysis showed no significant differences between the autograft (SFI − 60.19) and PCL + MSC groups (SFI, − 67.06) (*p* > 0.05). However, significant differences were observed between the autograft (SFI, − 60.19) and PCL groups (SFI, − 74.98) (*p* = 0.02). After 11 weeks, significant differences were observed comparing the autograft (SFI, − 52.58) group with the PCL + MSC (TFI, − 67.30) (*p* = 0.02) and PCL (SFI, − 77.39) groups (*p* < 0.001). Significant differences were observed upon comparison of the results of the autograft (SFI, − 50.40) group with those of PCL + MSC (SFI, 65.12) (*p* = 0.03) and PCL (SFI, − 80.81) groups (*p* < 0.001) after 12 weeks. However, after 12 weeks, the analysis demonstrated superior results with the PCL + MSC group (SFI, 65.12) than those in the PCL (SFI − 80.81) group (*p* < 0.02). Thus, the autograft and PCL + MSC groups showed better functional motor recovery than the PCL group (Fig. 4a).

Regarding TFI evaluation, no significant differences were observed from TFI analysis performed after 11 weeks between the autograft (TFI, − 60.25) and the PCL + MSC groups (TFI, − 75.98) (*p* > 0.05) (Fig. 4b). In addition, the autograft group demonstrated better results (TFI, − 64.25) than those in the PCL group (TFI, − 82.81) (*p* = 0.004). Similarly, after 12 weeks, no significant differences were observed between the autograft (TFI, − 60.34) and PCL + MSC groups (SFI, − 72.69) (*p* > 0.05) (Fig. 4b). However, the autograft group (TFI, − 60.34) was superior to that of the PCL (TFI, − 82.04) group (*p* < 0.001). The PCL + MSC group demonstrated superior functional motor recovery compared to the PCL group.

**Fig. 5:**
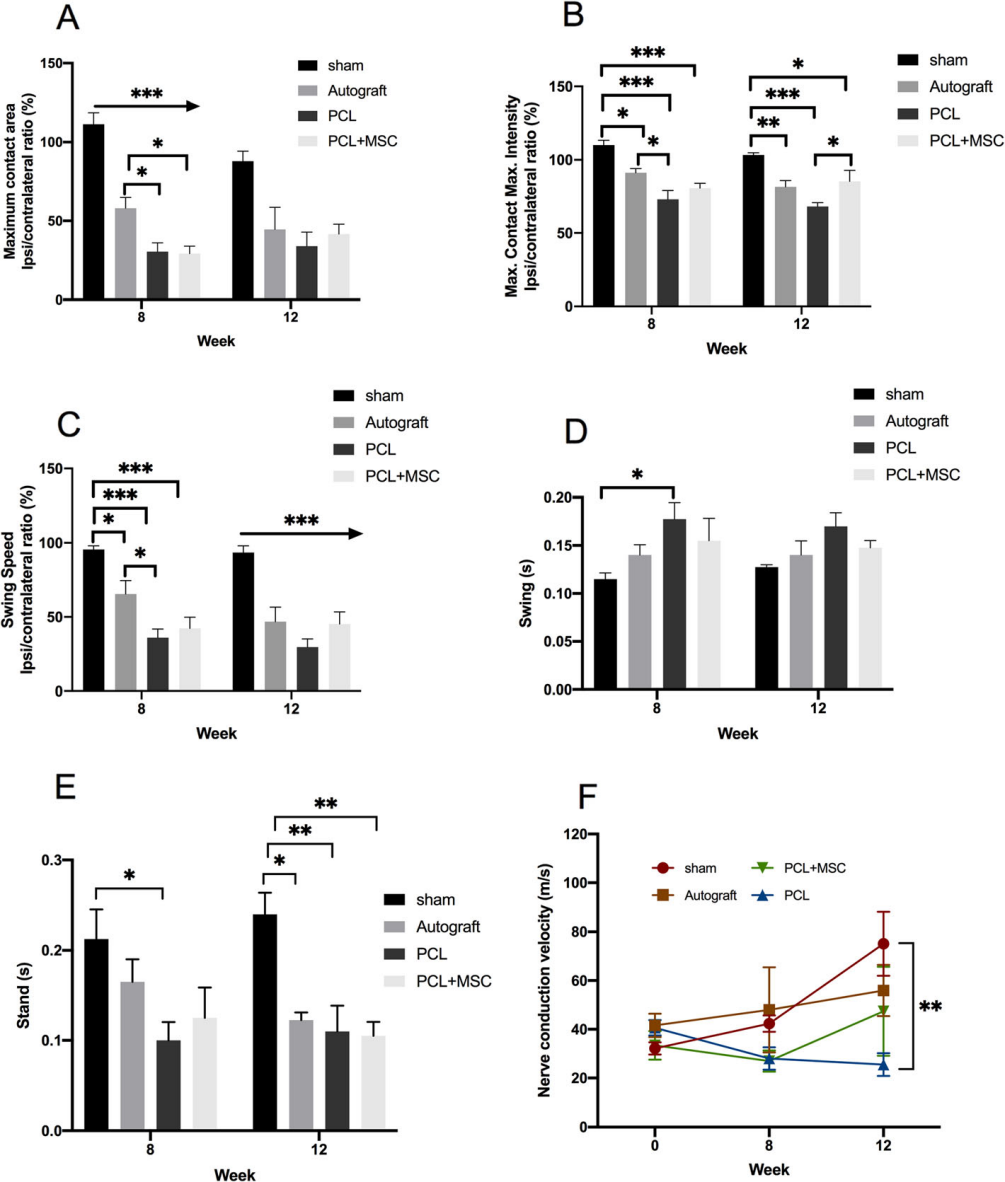
Gait analysis using the CatWalk platform and nerve conduction velocity (NCV) at 8 and 12 weeks in the experimental groups. **a** Maximum contact area. **b** Maximum contact intensity. **c** Swing speed. **d** Swing. **e** Stand time. **f** NCV (m/s). The values obtained are represented as mean ± SEM. *p* < 0.05*; *p* < 0.01**; *p* < 0.001***

After 8 and 12 weeks during gait analysis by Catwalk, the sham group demonstrated an improved maximum contact area in comparison with the contact area demonstrated by the autograft, PCL, and PCL + MSC groups (*p* < 0.001). The autograft group showed better values after 8 weeks than those in the PCL (*p* = 0.03) and PCL + MSC (*p* = 0.02) groups (Fig. 5a).

After 8 and 12 weeks, no significant differences were observed in the maximum contact intensity between the autograft and PCL + MSC groups (*p* > 0.05). However, the autograft group demonstrated superior results to that of the PCL group (*p* = 0.03). After 12 weeks, maximum contact intensity was significantly higher in the PCL + MSC group than in the PCL group (*p* = 0.04) (Fig. 5b).

After 8 and 12 weeks, no significant differences were observed in the swing speed between the autograft and PCL + MSC groups (*p* > 0.05). After 8 weeks, the autograft group demonstrated superior results to that of the PCL group (*p* = 0.02) (Fig. 5c).

After 8 and 12 weeks, no significant differences were observed in the swing values of the sham group when compared with the autograft and PCL + MSC groups (*p* > 0.01). However, after 8 weeks, the swing values of the sham group were superior to that of the PCL group (*p* = 0.01) (Fig. 5d).

After 8 and 12 weeks, no significant differences were observed during the analysis of spontaneous locomotion after 8 weeks with respect to the stand time (s) among the sham, autograft, and PCL + MSC groups (*p* > 0.05). However, the sham group demonstrated a better stand than the PCL group (*p* = 0.019). After 12 weeks, the sham group demonstrated an improved stand compared with the autograft (*p* = 0.014), PCL (*p* = 0.006), and PCL + MSC (*p* = 0.004) groups (Fig. 5e).

### NGCs multi-functionalized showed electrophysiological recovery

After 8 weeks, no significant differences were observed in the conduction velocity of regenerated nerves among the sham (42.35 m/s), autograft (47.99 m/s), PCL (28.02 m/s), and PCL + MSC (26.98 m/s) groups (*p* > 0.05). After 12 weeks, no significant differences in the NCV were observed among the sham (75.08 m/s), autograft (55.96 m/s), and PCL + MSC (47.37 m/s) groups (*p* > 0.05). The NCV was significantly reduced in the PCL group when compared to control (25.50 m/s) (*p* = 0.001). However, no significant differences were observed among the autograft and PCL + MSCs than the control group (*p* > 0.05). These findings demonstrate an increase in NCV in the autograft and PCL + MSC groups, as shown in Fig. 5f.

### Morphometric analysis of regenerated nerves

**Fig. 6:**
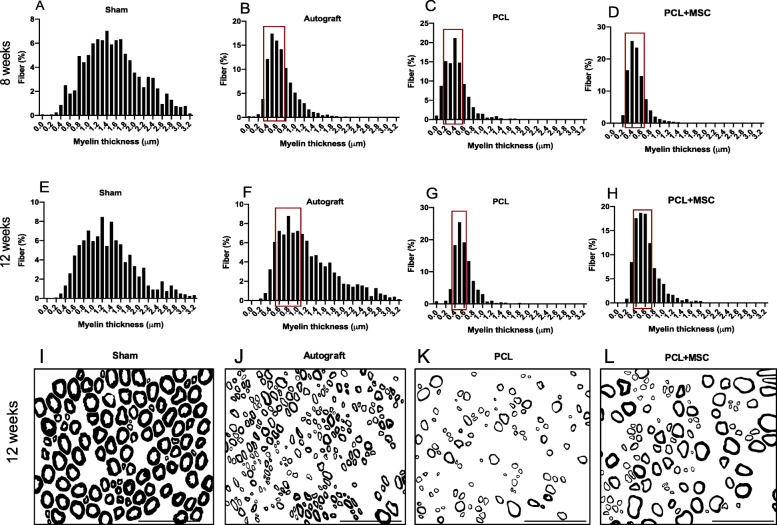
Frequency distribution of the myelin thickness in the experimental groups. **a**–**d** Values were obtained after 8 weeks following the lesion in the experimental groups. **e**–**h** Values were obtained after 12 weeks following the lesion in the experimental groups. At 12 weeks, myelin thickness was superior in the PCL + MSC group compared with the PCL group. Red boxes highlight frequency intervals with better percentages among the autograft, PCL, and PCL + MSC groups. **i**–**l** Tendency to increase myelinated axons with larger diameters in GA and GPCL + MSCs compared to GPCL in 12 weeks was observed. Scale bar = 50 μm

Myelin thickness measures with superior percentages after 8 weeks are as follows: sham (myelin sheath thickness 1.2 to 1.7 μm, mean 1.54 ± 0.01), autograft (myelin sheath thickness 0.4 to 0.7 μm, mean 0.71 ± 0.01), PCL (myelin sheath thickness 0.2 to 0.5 μm, mean 0.43 ± 0.01), and PCL + MSC (myelin sheath thickness 0.3 to 0.6 μm, mean 0.50 ± 0.01) (Fig. 6a–d). After 12 weeks, the myelin thickness measures were as follows: sham (myelin sheath thickness 1.1 to 1.5 μm, mean 1.35 ± 0.01), autograft (myelin sheath thickness 0.6 to 1.0 μm, mean 1.22 ± 0.01), PCL (myelin sheath thickness 0.4 to 0.7 μm, mean 0.59 ± 0.01), and PCL + MSC (myelin sheath thickness 0.4 to 0.7 μm, mean 0.63 ± 0.01) as shown in Fig. 6e–h. Myelination was observed in GPCL + MSCs close to GA in 12 weeks (Fig. 6i–l).

**Fig. 7:**
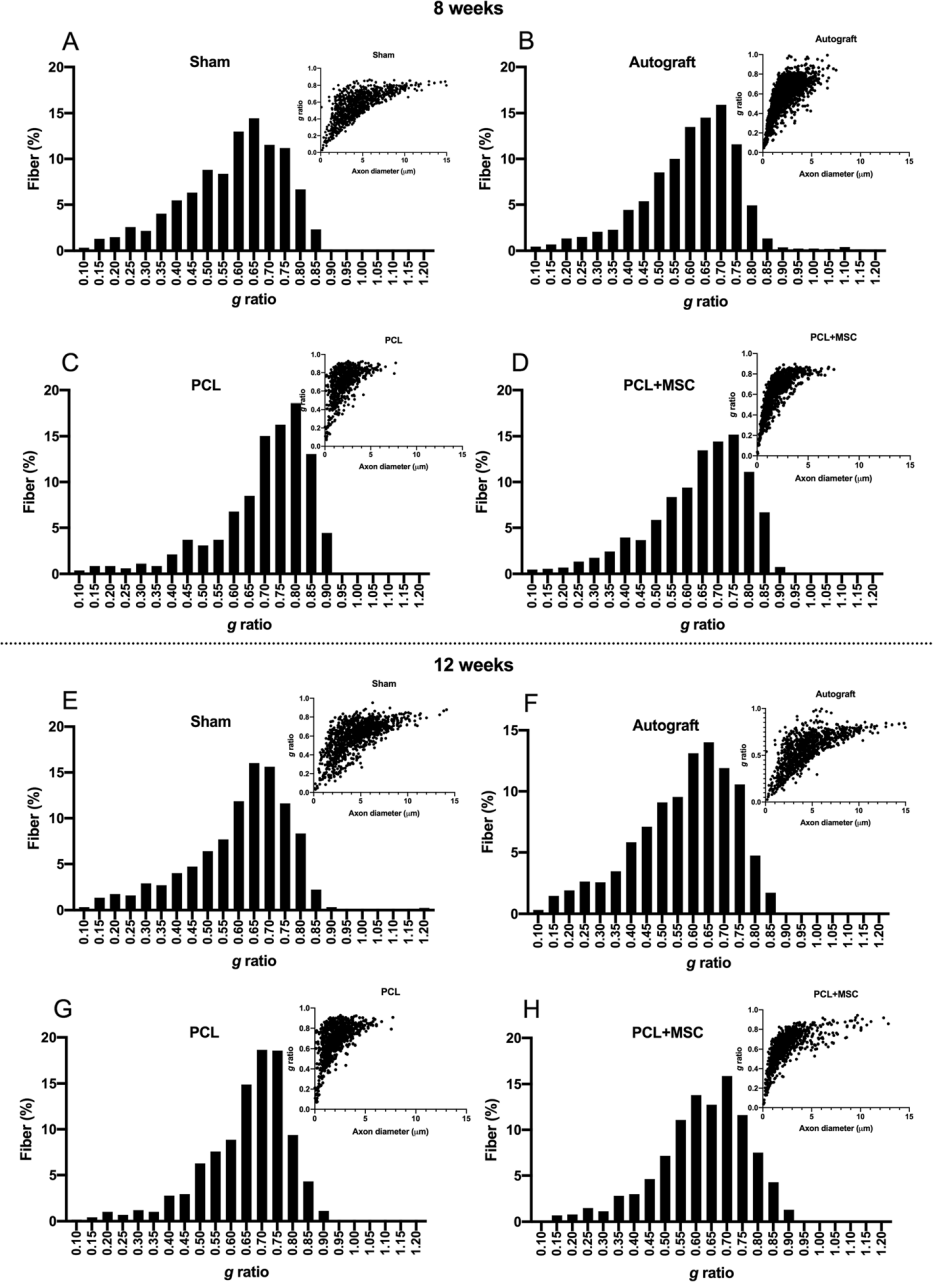
Frequency distribution of “g” ratio and dot plot of “g” ratio/axon diameter in the experimental groups. **a**–**d** Values were obtained 8 weeks after the lesion in the experimental groups. **e**–**h** Values were obtained 12 weeks after the lesion in the experimental groups. Note the shift towards an increase in diameter of the myelinated axon in the autograft and PCL + MSC groups when compared to the PCL group after 8 and 12 weeks

Measures of the “g” ratio with superior percentages after 8 weeks are as follows: sham (0.6 to 0.65 μm, mean 0.58 ± 0.01), autograft (0.65 to 0.7 μm, mean 0.60 ± 0.01), PCL (0.75 to 0.80 μm, mean 0.70 ± 0.01), and PCL + MSCs (0.65 to 0.75 μm, mean 0.63 ± 0.01) (Fig. 7a–d). After 12 weeks, they were as follows: sham (0.65 to 0.70 μm, mean 0.60 ± 0.01), autograft (0.60 to 0.65 μm, mean 0.57 ± 0.01), PCL (0.75 to 0.8 μm, mean 0.68 ± 0.01), and PCL + MSCs (0.6 to 0.7 μm, mean 0.62 ± 0.01). The correlation between the “g” ratio and myelinated axon diameter showed a shift towards a higher number of axons exhibiting close to normal myelination in the autograft and PCL + MSC groups after 12 weeks, as shown in Fig. 7e–h.

### NGCs multi-functionalized enhanced p75^NTR^ expression and preservation of Schwann cell reactivity

**Fig. 8:**
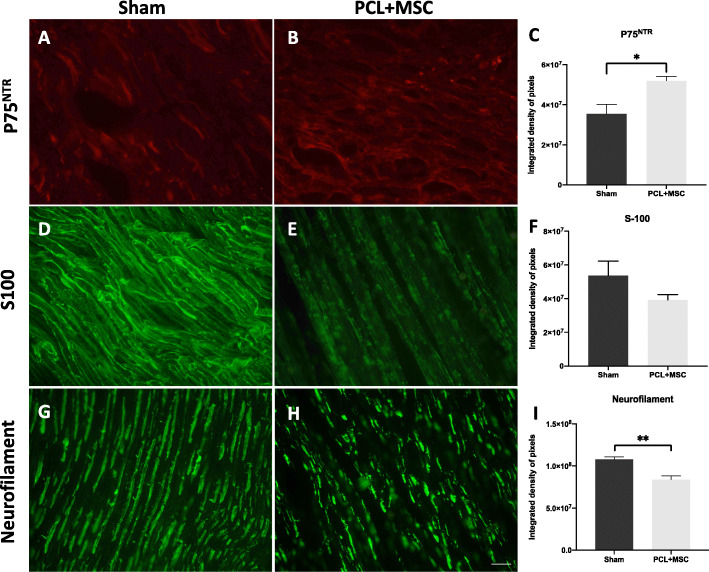
Fluorescence intensity analysis of p75^NTR^, S100, and neurofilament on sciatic nerve at 30 days in both sham and PCL + MSC groups. **a**–**c** Expression of the receptor p75^NTR^ in the proximal region of the nerve and integrated pixel density analysis. **d**–**f** Expression of the Schwann cell marker S-100 in the proximal region of the nerve and integrated pixel density analysis. **g**–**i** Expression of neurofilament in the proximal region of the nerve and integrated pixel density analysis. The values of the integrated pixel density are represented as mean ± SEM. *p* < 0.05*; *p* < 0.01**; *p* < 0.001***. Scale bar: 50 μm

Immunoreactivities of p75^NTR^, Schwann cells (S-100 expression), and neurofilament were evaluated in response to sciatic nerve regeneration 30 days after lesion formation in both sham and PCL + MSC groups. Immunoreactivity of the p75^NTR^ receptor was higher in the PCL + MSC group (5.1 × 10^7^ ± 0.37 × 10^7^) than in the sham group (3.5 × 10^7^ ± 0.83 × 10^7^) (*p* = 0.03) (Fig. 8a–c). No significant differences in the immunoreactivity of S-100 were observed between the sham (5.3 × 10^7^ ± 0.14 × 10^7^) and PCL + MSC groups (3.9 × 10^7^ ± 0.55 × 10^7^) (*p* < 0.05) (Fig. 8d–f). The reactivity of Schwann cells was preserved (Fig. 8b). The immunoreactivity of NF was superior in the sham group (10.7 × 10^7^ ± 0.07 × 10^7^) than in the PCL + MSC group (8.3 × 10^7^ ± 0.1 × 10^8^) (*p* = 0.001) (Fig. 8g-i).

### NGCs multi-functionalized stimulated expression of neurotrophic factors

**Fig. 9:**
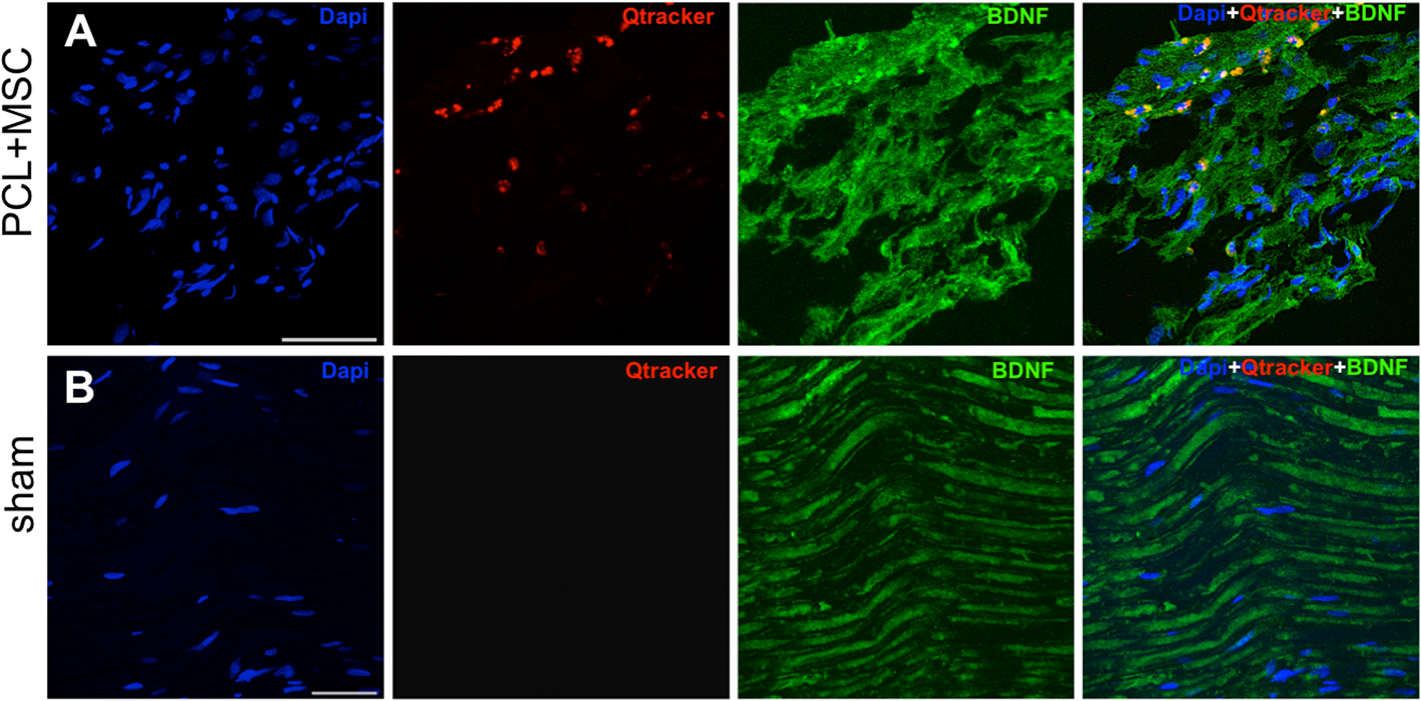
Representative micrographs of BDNF immunolabeling on sciatic nerve at 30 days, in the Sham and PCL + MSC groups. **a** Representative images of triple labeling with anti-BDNF (green), DAPI (blue), and Qtracker® qdot655 (red) in the proximal region of the nerve. Co-localization of cells marked with qdot655 and nuclei with DAPI was observed, indicating the presence of living cells within the internal structure of the NGC. **b** BDNF immunolabeling on sham group. Cells labeled with qdot655 are absent. Scale bar: 25 μm

**Fig. 10:**
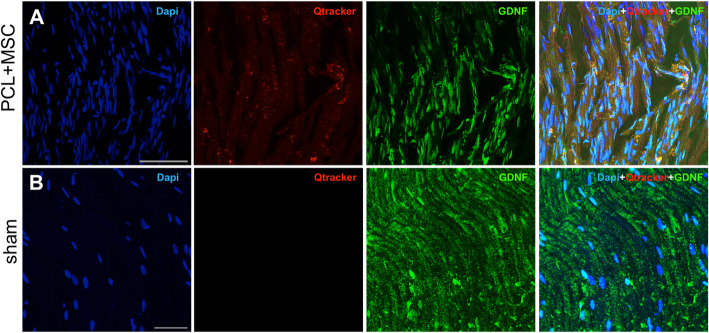
Representative micrographs of GDNF immunolabeling on sciatic nerve at 30 days, in the sham and PCL + MSC groups. **a** Representative images of triple labeling with anti-GDNF (green), DAPI (blue), and Qtracker® qdot655 (red) in the proximal region of the nerve. It is important to note the reactivity of GDNF close to canine AdMSCs (**b**). GDNF immunolabeling on sham group. Scale bar: 25 μm

Immunoreactivity against BDNF and GDNF was characterized in regenerating nerves obtained inside the NGC in the sham group and PCL + MSC group 30 days after the lesion. The positive expression of BDNF and GDNF is shown in Figs. 9a and 10a. An increase in the intensity of the immunostaining for BDNF and GDNF was observed from the proximal region of the nerve in the PCL + MSC group compared with the sham group. The intensity was stronger in the proximal region, indicating that the PCL biomaterial functionalized with MSCs positively co-stimulated the production of neurotrophins BDNF and GDNF.

### Canine AdMSCs engrafted after nerve repair

Serial histological sections were obtained 30 days following nerve repair with NGC multi-functionalized for detection of the labeled cells. Canine AdMSCs labeled with qdot655 were observed inside the NGC, in the proximal stump, confirming the survival of these cells for at least 4 weeks in vivo (Fig. 9a and 10a). However, cells were not observed in the distal stumps or in regions not located in the proximity of NGC, indicating that the cells were possibly retained around the application site. In addition, the labeled cells showed co-localization with the regions that demonstrated positive immunostaining for BDNF and GDNF (Fig. 9a and 10a).

### NGCs multi-functionalized enhanced upregulation of the expression of BDNF, GDNF, and HGF in the spinal cord

**Fig. 11:**
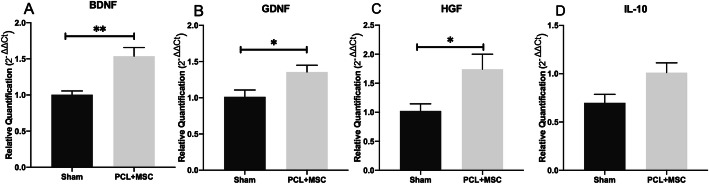
Gene expression in the ipsilateral spinal cord at 30 in both sham and PCL + MSC groups. **a** Relative expression of BDNF. **b** GDNF. **c** HGF. **d** IL-10. Samples were tested with two reference genes, β2-microglobulin and hypoxanthine phosphoribosyl transferase (HPRT). Data are represented as mean ± SEM. *p* < 0.05*; *p* < 0.01**; *p* < 0.001***

Gene expression of the neurotrophins BDNF, GDNF, and HGF was evaluated 30 days after nerve repair in both sham and PCL + MSC groups. The expression of BDNF was significantly higher in the PCL + MSC group (1.53 ± 0.11) than in the sham group (1.00 ± 0.05) (*p* = 0.006). The expression of GDNF was significantly higher in the PCL + MSC group (1.35 ± 0.09) than in the sham group (1.01 ± 0.09) (*p* = 0.04). The expression of HGF was significantly higher in the PCL + MSC group (1.74 ± 0.25) than in the sham group (1.02 ± 0.12) (*p* = 0.04), as shown in Fig. 11a–c. No significant differences in IL-10 expression were observed in the PCL + MSC group (1.01 ± 0.10) when compared with the sham group (0.69 ± 0.17) (*p* > 0.05) (Fig. 11d).

## Discussion

A tissue engineering approach that integrates NGCs, cells, and growth factors mimicking native tissues shows promise for restoring the damage in nervous tissue [11, 12, 63–66]. Exogenous application of growth-promoting is beneficial for nerve regeneration [64–66]. Nevertheless, this approach raises concerns regarding short duration, dosage, and high cost [64–66]. To overcome these limitations, studies indicated that AdMSCs secrete a complex mix of factors that are capable of promoting myelination, regenerating nerve fibers, exerting neuroprotective effects, stimulating angiogenesis, and modulating the inflammatory environment [34–36]. Although the criteria are not fully defined for canine AdMSC characterization, the cells used in this study exhibited a comparable in vitro profile in line with previously published studies [31, 48, 49, 63]. The neuroprotective effects of MSCs are primarily associated with the production of BDNF, GDNF, nerve growth factor (NGF), insulin-like growth factor (IGF), and HGF [30, 34, 35, 67–71]. It is also influenced by immunoregulatory mechanisms associated with interleukin 10 (IL-10), prostaglandin E2 (PGE2), indoleamine 2,3-dioxygenase (IDO), HGF, and transforming growth factor-beta (TGF-β) [36, 72–74]. Here, we evaluated the neurogenerative capacity of NGCs fabricated by 3D printing and multi-functionalized with canine AdMSCs using HFB as cell scaffold to restore the damage caused by critical sciatic nerve injury in rats.

The inflammatory environment during Wallerian degeneration is indispensable for axonal regeneration and is characterized by a significant production of tumor necrosis factor alpha (TNF-α) and IFN-γ by Schwann cells and fibroblasts during the first 14 days following injury. This leads to the recruitment of inflammatory cells [75–78]. These infiltrated immune cells lead to a rapid clearance of myelin and facilitate nerve regeneration [79]. In this study, canine AdMSCs demonstrated constitutive expression of BDNF, GDNF, HGF, and IL-10. Furthermore, direct stimulation with IFN-γ resulted in the upregulation of the expression of BDNF, GDNF, HGF, and IL-10. Neurotrophic factors BDNF, GDNF, and HGF are powerful molecules that act synergistically and influence nerve-muscle synapsis, neuronal survival, proliferation of Schwann cells, and axonal regeneration [80–82]. IL-10 is a cytokine involved in the restoration of tissues via the regulation of inflammatory responses, extracellular matrix production, fibroblast functions, and angiogenesis [83, 84]. Studies have shown that IFN-γ is a key player in activating the immunomodulatory function of murine and human MSCs through the production of several factors, including HGF and IL-10, which is consistent with the results of our study [85, 86]. More importantly, through inflammation enriched with proinflammatory cytokines such as IFN-γ, MSCs could be activated or primed, and the upregulation of major histocompatibility complex class I (MHC-I) can lead to improved survival [37].

Versatile NGCs should demonstrate biocompatibility along with the modulation of the cellular environment that permits cell adhesion, axonal branching, and revascularization [11]. PCL polymer has been used for the fabrication of hollow NGCs via conventional manufacturing methods, showing positive regeneration in rats [27, 28, 87]. Rapid prototyping methods such as FFF 3D printing for NGC fabrication enable the control of porosity, architecture, reproducibility, customizability, and scalability [20]. Herein, 3D-printed NGCs showed characteristics such as mechanical strength, macroporosity, and adequate geometry, in addition to biocompatibility. The fabrication of NGC with a customized architecture has been demonstrated using 3D-printing technology with a PCL polymer in few studies [21–23]. Our design of 3D printed-NGC was based on controlled parameters reported to maintain nutrients diffusion such as porosity (sizes of 125–550 μm) and wall thickness (size of 600 μm) not affecting mechanical properties [22, 80, 88]. Internal diameter tailored to the nerve size (< 2 mm) to avoid compression and support diffusion [13, 17]. In addition, the construct was designed based on lesion size (length of 1.5 cm) suitable to bridging the gap tension free [7].

Long-gap sciatic lesions in rats (such as a 10-mm gap) are considered critical, with only 10% axons effectively regenerating into the NGC [11, 55]*.* In our study, NGCs multi-functionalized showed positive results for functional motor recovery, being superior in the TFI. After 8 weeks, the autograft group showed better results, following the PCL + MSC group. In addition, Catwalk analysis demonstrated an increase in the duration of the support phase (stand time), contact intensity, swing time, and velocity after 8 weeks in the PCL + MSC group compared with the PCL group. Previous studies have demonstrated functional motor recovery after a combination of allogenic MSCs and PCL-NGCs were implanted in short gaps with lengths of 3–10 mm [26, 89, 90]. In contrast, allogeneic Schwann cells, MSCs, and polylactic acid (PLA) NGCs demonstrated functional recovery after 8 weeks with gaps of 15 mm. However, the SFI was lower than that observed in our study [91]. Using the 3D printing approach, NGCs were customized using composite material to be implanted in a short gap of 4 mm in mice. However, only the sensitive function of the nerve was evaluated compared to our study [23]. In our study, our critical lesion level reflects a better clinical setting without endogenous regeneration influence. Moreover, retraction of stumps could increase the gap and negatively influence functional recovery [11, 55]*.* In sciatic nerve models, muscle contractures can be observed that influence negatively on SFI results, which is not observed with IFT method [55, 92]*.* In addition, variability in recovery between SFI and TFI can result from complexity of mixed fibers of sciatic nerve that would have inappropriate motor-motor as well motor-sensory connections [92]*.*

Electrophysiological and histological evaluations are complementary techniques used for the examination of nerve regeneration. In our study, the PCL + MSC and autograft groups showed better results in NCV after 12 weeks, indicating the presence of myelinated axons. In addition, the morphometric analysis demonstrated improvements in myelin thickness in the PCL + MSC group compared with the PCL group after 8 and 12 weeks. The correlation between the “g” ratio and myelinated axon diameter showed a shift towards a higher number of axons with presence of myelination after 12 weeks. Previous studies have shown improvements in the histological parameters of short-gap defects after a combination of MSCs and PCL implanted in gaps of 5–10 mm [26, 89, 90]. In long-gap defects, recovery of NCV and the presence of the highest number of myelinated axons were observed with allogeneic MSCs plus PLA-NGC after 6 weeks [93]. However, the NCV obtained in that study was lower (about 40 m/s) than that observed in our study [93]. Other studies have demonstrated NCV and morphometric recovery after 8 and 12 weeks following the use of MSCs and Schwann cells applied in a PLA-NGC or an acellular nerve allograft. Despite positive functional recovery, the NCVs (12.45 and 17 m/s, respectively) were slower than those of the PCL + MSC group observed in our study (47.37 m/s) [91, 94]. A limitation of NCV is that poor correlation with the SFI is observed, because both parameters measure different aspects of nerve regeneration, explaining variation between NCV and SFI results at 12 weeks after nerve repair [95].

In our study, in vivo parameters (SFI, TFI, Catwalk analysis, NCV, and morphological results) were compared with an additional group, consisting of NGC multi-functionalized with rat AdMSCs embedded in HFB (PCL+ rMSCs). On functional and electrophysiological evaluation, PCL+ rMSCs and canine PCL + MSCs presented similar results without meaningful differences between them (*p* > 0.05) (Additional file 5: Figure S2; Additional file 6: Figure S3). Morphological analysis showed similar frequency in diameter fiber and axon diameter (Additional file 7: Figure S4; Additional file 8: Figure S5); however, myelin thickness was superior in canine AdMSCs when compared to rat AdMSCs at 8 and 12 weeks (Additional file 9: Figure S6; Additional file 10: Figure S7; Additional file 11: Figure S8). These findings are in line with several studies that verified regenerative potential of rat AdMSCs [26, 89–91, 94]. On the other hand, few studies have shown peripheral nerve regenerative potential of canine AdMSCs [63].

Nerve regeneration is strongly influenced by a pro-regenerative microenvironment [80]. Neurotrophins act selectively in high-affinity tropomyosin receptor kinases (trk) and low-affinity receptors p75^NTR^ and are expressed in Schwann cells and growth cones in regenerating nerves [9, 80, 96]. Immunolabeling results indicated a positive expression of BDNF and GDNF in association with p75^NTR^ in the PCL + MSC group after 30 days. Our results are comparable to those observed in previous studies in which nerve regeneration was frequently associated with the production of BDNF and GDNF during the first few weeks after MSC transplantation [34, 35, 68, 70, 74, 97]. BDNF and GDNF activate several in vivo and in vitro pathways associated with nerve regeneration, formation of nerve-muscle synapses, neuronal survival and proliferation, and survival of Schwann cells [80, 96]. Activation of the BDNF/p75^NTR^ pathway instead of BDNF/trkB plays an important role in the activation and differentiation of Schwann cells as well as in myelination [80, 96, 98]. Previous studies conducted with polymeric NGC (6- and 10-mm gaps) and allogeneic MSCs demonstrated the proliferation of Schwann cells and increased expression of neurotrophic receptors after 2 and 8 weeks, respectively [88, 99].

Schwann cells are crucial for axonal branching and myelin production [9, 80]. The reactivity of Schwann cells and the organization of the cytoskeleton were evaluated using S-100 and neurofilament markers. Reactivity of S-100 was increased 30 days after nerve repair with NGC plus MSCs. NF immunostaining showed values that were close to those of the sham group. Previous studies observed S-100 expression in rats treated with allogeneic and xenogeneic AdMSCs after nerve injury [26, 63, 99]. Co-expression of S100 and neurotrophin receptors (p75^NTR^ and trks) was detected in axonotmesis or neurotmesis experiments after MSC transplantation in rats [88, 99].

Paracrine and regenerative effects depend on AdMSC engraftment [31, 37, 73]. In our study, AdMSCs embedded in HFB survived for 30 days after transplantation into the NGC and were co-localized with BDNF and GDNF. Similarly, human AdMSCs transplanted after root avulsion in rats increased neuronal survival mediated by BDNF, GDNF, and HGF. Human MSCs were able to engraft and survive in the lesion area for at least 14 days [74]. The results of another study demonstrated the co-expression of BDNF and allogeneic MSCs positive for green fluorescent protein after 60 days, indicating the continuous activation of these cells [25]. The main mechanism of HFB is the adhesion ability allowing support and viability of the cells for several weeks [39–44]. In our study, the application of HFB acted synergistically with the MSCs for enhancing pro-regenerative effects and thereby contributing to nerve regeneration. As shown by immunofluorescence, canine AdMSC in co-localization with the BDNF, GDNF, and p75^NTR^ indicates that the persistence of viable cells into the microenvironment of lesion was crucial for the neurotrophic factor maintenance released by the cells. Previous studies showed that the use of HFB for reimplantation of ventral nerve roots lesioned or end-to-end nerve cooptation, proved cell support capacity, adhesion for axonal regeneration and neuroprotection [40–46].

Degeneration, loss of inhibitory and excitatory synapses, formation of glial scarring, and excitotoxicity are observed in neuronal bodies following peripheral nerve injury [100, 101]. Regulation of several genes related to cell survival and axonal growth indicates changes in the pro-regenerative status of motor neurons [100]. Here, the upregulation of the expression of BDNF, GDNF, and HGF was detected after 30 days in the PCL + MSC group, indicating a pro-regenerative response in the ventral horn of the spinal cord. In a ventral root injury model, MSC transplantation increased BDNF expression 2 weeks after injury [97]. The results of previous studies did not demonstrate the expression of neurotrophic factors in spinal cord lesions following MSC transplantation in rats or humans [101–103]. We assumed that the paracrine production of pro-regenerative factors by MSCs might result in the formation of a gradient of molecules secreted throughout peripheral nerves and influence the spinal cord to contribute to nerve regeneration.

## Conclusion

The tissue engineering approach for nerve regeneration based on 3D-printed NGCs multi-functionalized with canine AdMSCs embedded in HFB showed positive functional and electrophysiological locomotor recovery after 8 and 12 weeks following critical experimental injury. In addition, it shifted to a pro-regenerative profile mediated by neurotrophic factors during the first 4 weeks in the microenvironment of nerves and the spinal cord, thereby improving functional recovery. Although combinatorial approaches for the treatment of PNI injuries are highly desirable, further studies are necessary to overcome the autograft technique analyzing several geometric parameters with 3D printing, as well as direct priming of MSCs and neurotrophic factors to enhance nerve regeneration.

## Supplementary Information

**Additional file 1: Table S1.** Primers used for RT-qPCR in canine AdMSCs.

**Additional file 2: Table S2.** Primary antibodies used for immunofluorescence.

**Additional file 3: Table S3.** Primers used for RT-qPCR in the spinal cord.

**Additional file 4: Figure S1.** Immunophenotyping and differentiation potential of canine AdMSCs. (a) Adipogenic differentiation at 14 days. Control of adipogenic differentiation (inserted in a). (b) Osteogenic differentiation at 21 days. Control of osteogenic differentiation (inserted in b). (c) Chondrogenic differentiation at 21 days. Cell micromass after differentiation. (inserted in c). (d) Phenotypic characterization. Gate in canine AdMSCs (cell size versus granularity). (e-h) histograms (negative control - blue; percentage of expression of surface markers - green). (e) CD90: 94,03%; (f) CD45: 2,10%; (g) CD71: 4,71%; (h) CD34: 0,58%. Scale bar, 50 μm.

**Additional file 5: Figure S2.** Sciatic nerve functional index (SFI) and tibial functionality index (TFI) during 12 weeks in the Sham, autograft, PCL, and PCL+ rMSC groups. (a) SFI, (b) TFI. The line red represents the values of the PCL + MSC group. The values were obtained weekly and are represented as mean ± SEM. *p* < 0.05*; *p* < 0.01**; *p* < 0.001***.

**Additional file 6: Figure S3.** Gait analysis using the CatWalk platform and nerve conduction velocity (NCV) at 8 and 12 weeks, comparing the Sham, autograft, PCL, and PCL + rMSC groups. (a) Maximum contact area, (b) maximum contact intensity, (c) swing speed, (d) swing, (e) stand time, and (f) NCV (m/s). The red bar and line characterize the PCL + MSC group. The values obtained are represented as mean ± SEM. p < 0.05*; p < 0.01**; p < 0.001***.

**Additional file 7: Figure S4.** Frequency distribution of myelinated fiber diameter at 8 (a-e) and 12 weeks (f-j) after the lesion in the sham, autograft, PCL, PCL + rMSC and PCL + MSC groups. Similar values in the frequency distribution of myelinated fiber diameter were observed among PCL + rMSC and PCL + MSC groups at 8 and 12 weeks. The red boxes highlight frequency intervals with better percentages among autograft, PCL and PCL + MSC groups.

**Additional file 8: Figure S5.** Frequency distribution of axon diameter at 8 (a-e) and 12 weeks (f-j) after the lesion in the sham, autograft, PCL + rMSC and PCL + MSC groups. Similar values in the frequency distribution of axon diameter were observed among PCL + rMSC and PCL + MSC groups at 8 and 12 weeks. Red boxes highlight frequency intervals with better percentages among autograft, PCL and PCL + MSC groups.

**Additional file 9: Figure S6.** Frequency distribution of the myelin thickness in the Sham, autograft, PCL, PCL + rMSC and PCL + MSC groups at 8 weeks (a-e) and 12 weeks (f-j). At 12 weeks, myelin thickness was superior in the PCL + MSC group compared with the PCL and PCL + rMSC groups. Red boxes highlight frequency intervals with better percentages among the groups.

**Additional file 10: Figure S7.** Frequency distribution of “g” ratio and dot plot of “g” ratio/axon diameter thickness in the Sham, autograft, PCL, PCL + rMSC and PCL + MSC groups at 8 weeks (a-e). Note the shift towards an increase in the diameter of the myelinated axon in the autograft and PCL + MSC groups when compared to the PCL and PCL + rMSC groups at 8 weeks.

**Additional file 11: Figure S8.** Frequency distribution of “g” ratio and dot plot of “g” ratio/axon diameter thickness in the Sham, autograft, PCL, PCL + rMSC and PCL + MSC groups at 12 weeks (a-e). At 12 weeks, frequency distribution was similar in PCL + MSC and PCL + rMSC groups.

## Data Availability

The datasets used and/or analyzed during the current study are available from the corresponding author upon request.

## Abbreviations

PNS: Peripheral nervous system
NGC: Nerve guidance conduits
PGA: Polyglycolic acid
PCL: Polycaprolactone
3D: Tridimensional
MSCs: Mesenchymal stromal cells
AdMSCs: Mesenchymal stromal cells derived from adipose tissue
HFB: Heterologous fibrin biopolymer
Sham: Sham group
PCL + MSCs: Polycaprolactone nerve guidance conduits plus canine AdMSCs
SFI: Sciatic functional index
TFI: Tibial functional index
NCV: Nerve conduction velocity
RT-qPCR: Real-time PCR
DMEM: Dulbecco’s modified Eagle’s medium
FBS: Fetal bovine serum
IFN-γ: Interferon-gamma
RNA: Ribonucleic acid
cDNA: Complementary DNA
BDNF: Brain-derived neurotrophic factor
GDNF: Glial cell-derived neurotrophic factor
HGF: Hepatocyte growth factor
IL-10: Interleukin 10
GAPDH: Glyceraldehyde-3-phosphate dehydrogenase
HPRT: Hypoxanthine phosphoribosyltransferase
FFF: Fused filament fabrication
cMAP: Compound muscle action potentials
NF: Neurofilament H
p75^NTR^: p75 neurotrophin receptor
CY: Cyanine
PBS: Phosphate buffer
S100: Calcium-binding protein
NGF: Nerve growth factor
IGF: Insulin-like growth factor
bFGF: Basic fibroblast growth factor
VEGF: Vascular endothelial growth factor
IDO: Indoleamine 2,3-dioxygenase
TGF-β: Transforming growth factor-beta
TNF-α: Tumor necrosis factor alpha
MHC-I: Major histocompatibility complex class I
trk: High-affinity tropomyosin receptor kinases
