# Supplementary material for: 3D-printed nerve guidance conduits multi-functionalized with canine multipotent mesenchymal stromal cells promote neuroregeneration after sciatic nerve injury in rats
Source: Stem Cell Res Ther. 2021 May 29;12:303. doi: 10.1186/s13287-021-02315-8 (PMC8164252; doi:10.1186/s13287-021-02315-8)
Supplement: Supplementary file 1 — Additional file 1: Table S1. Primers used for RT-qPCR in canine AdMSCs. [file 13287_2021_2315_MOESM1_ESM.docx]

| **Gene** | **Sequence (5’ –> 3’)** |
| --- | --- |
| BDNF | **(*F*) GTGTCGAAAGGCCAACTGAAG** |
|  | **(*R*) CGTGTAACCCATGGGATTGC** |
| GDNF | **(*F*) GGTTTGCTACAGCCAGCAGTT** |
|  | **(*R*) CGCACCATGTTCAAAATCCA** |
| HGF | **(*F*) ATGGTTCTTGGCGTCATTGTT** |
|  | **(*R*) AATGCCAGGACGATTTGGAA** |
| IL-10 | **(*F*) CCCAGGATGGCAACTCTTCTC** |
|  | **(*R*) CGGGATGGTATTTTGCAGATC** |
| GAPDH | **(*F*) CATCAACGGGAAGTCCATCT** |
|  | **(*R*) TACTCACCACCAGCATCACC** |
| HPRT | **(*F*) CGGCTTGCTCGAGATGTGAT** |
|  | **(*R*) GCACACAGAGGGCTATGT** |

**Table S1.** Primers used for qPCR in canine AdMSCs

Brain derived neurotrophic factor (BDNF), glial cell-derived neurotrophic factor (GDNF), hepatocyte growth factor (HGF), interleukin 10 (IL-10), glyceraldehyde-3-Phosphate Dehydrogenase (GAPDH), hypoxanthine phosphoribosyltransferase (HPRT).
