# Supplementary material for: 3D-printed nerve guidance conduits multi-functionalized with canine multipotent mesenchymal stromal cells promote neuroregeneration after sciatic nerve injury in rats
Source: Stem Cell Res Ther. 2021 May 29;12:303. doi: 10.1186/s13287-021-02315-8 (PMC8164252; doi:10.1186/s13287-021-02315-8)
Supplement: Supplementary file 2 — Additional file 2: Table S2. Primary antibodies used for immunofluorescence. [file 13287_2021_2315_MOESM2_ESM.docx]

**Table S2.** Primary antibodies used for immunohistochemistry.

| **Antibody** | **Supplier** | **Host** | **Code** | **Concentration** |
| --- | --- | --- | --- | --- |
| S-100 | Abcam | Rabbit | AB868 | 1:500 |
| NF | Millipore | Rabbit | AB1989 | 1:200 |
| BDNF | Millipore | Rabbit | Ab1534 | 1:500 |
| GDNF | Millipore | Sheep | Ab5252p | 1:500 |
| p75^NTR^ | Santa Cruz | Goat | Sc-6188 | 1:250 |

Neurofilament H (NF), brain derived neurotrophic factor (BDNF), glial cell-derived neurotrophic factor (GDNF), p75 neurotrophin receptor (p75^NTR^).
