# Supplementary material for: 3D-printed nerve guidance conduits multi-functionalized with canine multipotent mesenchymal stromal cells promote neuroregeneration after sciatic nerve injury in rats
Source: Stem Cell Res Ther. 2021 May 29;12:303. doi: 10.1186/s13287-021-02315-8 (PMC8164252; doi:10.1186/s13287-021-02315-8)
Supplement: Supplementary file 3 — Additional file 3: Table S3. Primers used for RT-qPCR in the spinal cord. [file 13287_2021_2315_MOESM3_ESM.docx]

| **Gene** | **Sequence (5’ –> 3’)** |
| --- | --- |
| BDNF | **(*F*) GGCCCAACGAAGAAAACCAT** |
|  | **(*R*) AGCATCACCCGGGAAGTG** |
| GDNF | **(*F*) ACTTGGGTTTGGGCTACGAA** |
|  | **(*R*)CAGGAACCGCTACAATATCGAAA** |
| HGF | **(*F*) ATCGTGGCAATGGGAAAAAC** |
|  | **(*R*)GAACATGTGAGTCCAGACCTTGTT** |
| IL-10 | **(*F*) CCCAGGATGGCAACTCTTCTC** |
|  | **(*R*) CGGGATGGTATTTTGCAGATC** |
| IL-6 | **(*F*) CCCACCAGGAACGAAAGTCA** |
|  | **(*R*)**GCGGAGAGAAACTTCATAGCTGTT |
| ß2-microglobulin | **(*F*) GCCATCCACCGGAGAATG** |
|  | **(*R*)** GGTGGAACTGAGACACGTAGCA |
| HPRT | **(*F*) CGGCTTGCTCGAGATGTGAT** |
|  | **(*R*) GCACACAGAGGGCTATGT** |

**Table S3.** Primers used for qPCR in spinal cord.

Brain derived neurotrophic factor (BDNF), glial cell-derived neurotrophic factor (GDNF), hepatocyte growth factor (HGF), interleukin 10 (IL-10), interleukin 6 (IL-6), beta-2-microglobulin (ß2-microglobulin), glyceraldehyde-3-phosphate dehydrogenase (GAPDH), hypoxanthine phosphoribosyltransferase (HPRT).
