# Supplementary figures and images for: 3D-printed nerve guidance conduits multi-functionalized with canine multipotent mesenchymal stromal cells promote neuroregeneration after sciatic nerve injury in rats
Source: Stem Cell Res Ther. 2021 May 29;12:303. doi: 10.1186/s13287-021-02315-8 (PMC8164252; doi:10.1186/s13287-021-02315-8)

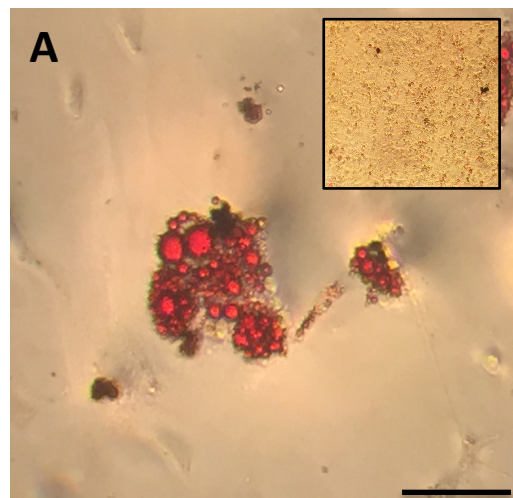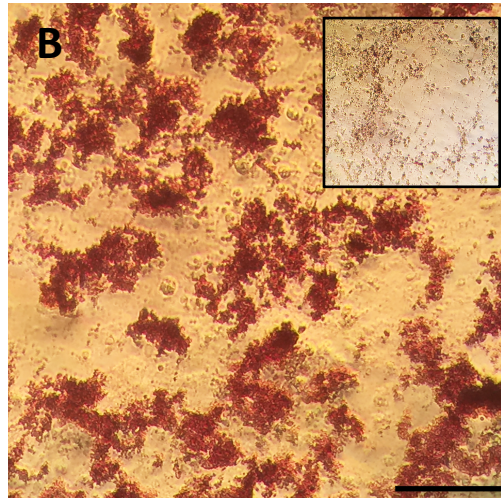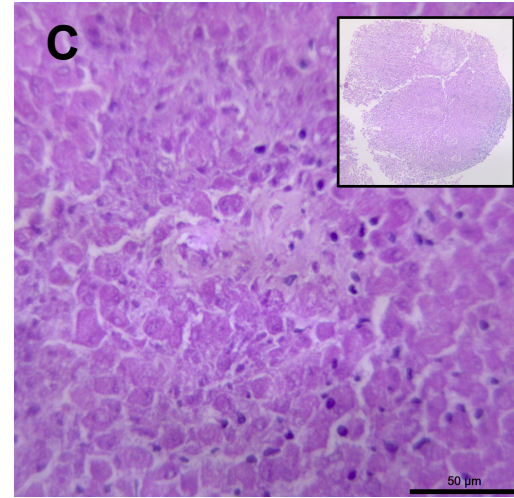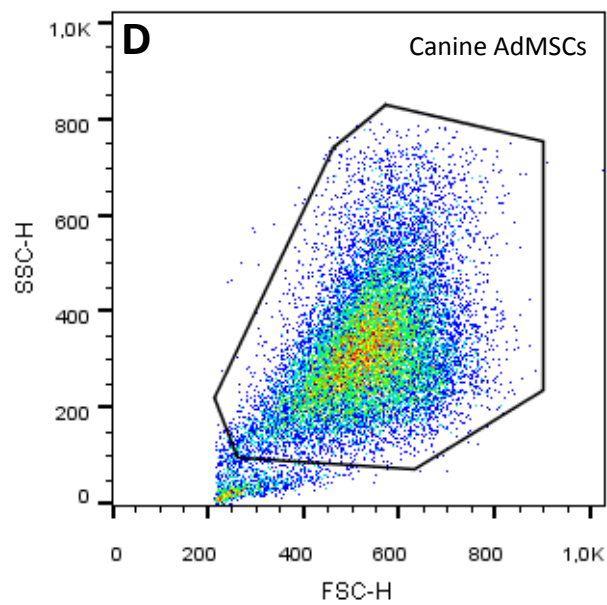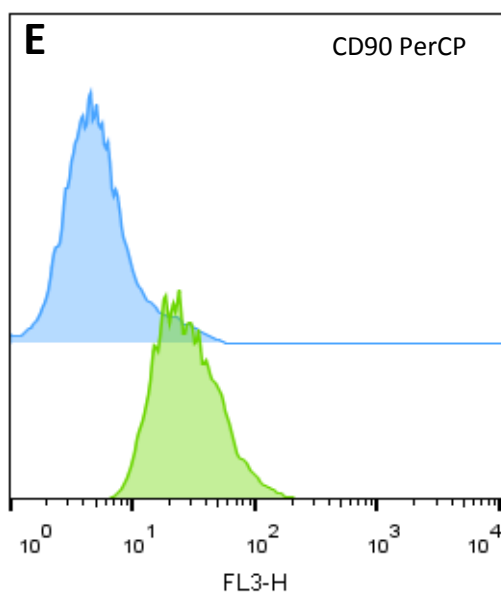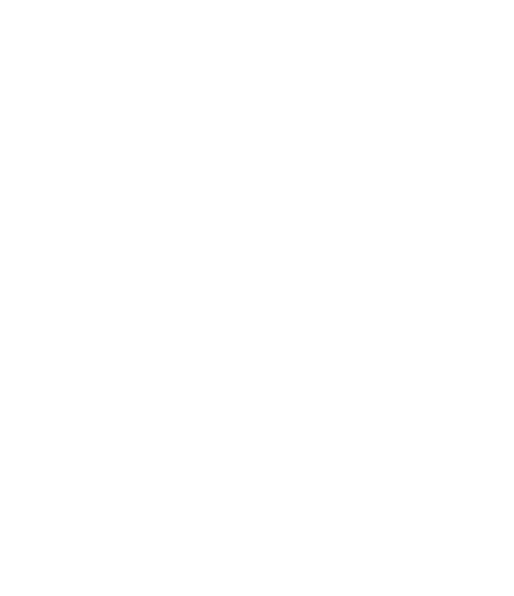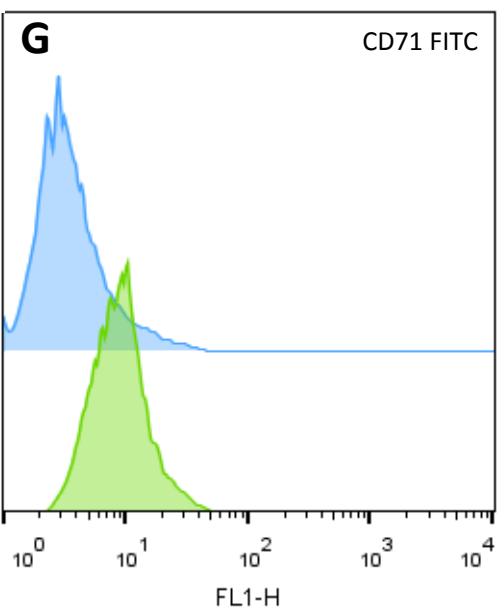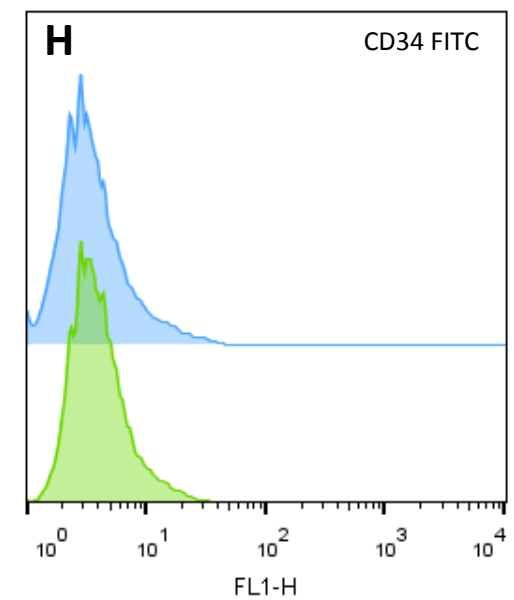

Supplement: Supplementary file 4 — Additional file 4: Figure S1. Immunophenotyping and differentiation potential of canine AdMSCs. (a) Adipogenic differentiation at 14 days. Control of adipogenic differentiation (inserted in a). (b) Osteogenic differentiation at 21 days. Control of osteogenic differentiation (inserted in b). (c) Chondrogenic differentiation at 21 days. Cell micromass after differentiation. (inserted in c). (d) Phenotypic characterization. Gate in canine AdMSCs (cell size versus granularity). (e-h) histograms (negative control - blue; percentage of expression of surface markers - green). (e) CD90: 94,03%; (f) CD45: 2,10%; (g) CD71: 4,71%; (h) CD34: 0,58%. Scale bar, 50 μm. [file 13287_2021_2315_MOESM4_ESM.pdf]

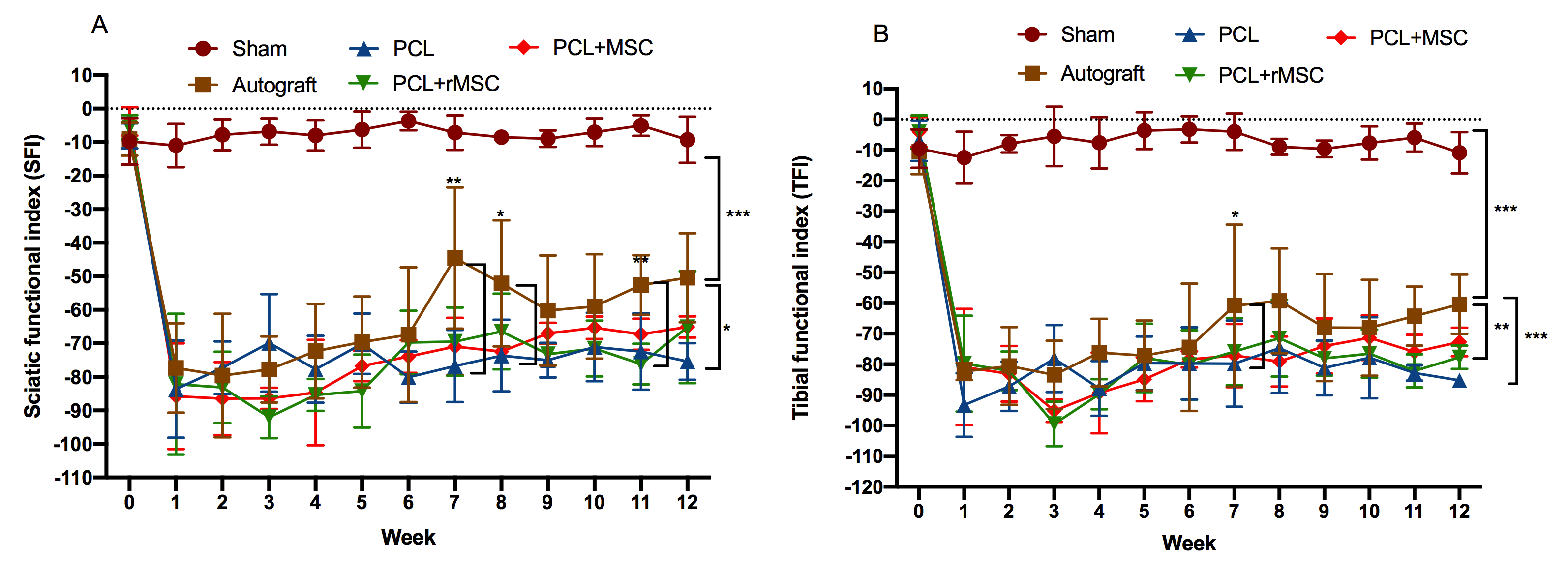

Supplement: Supplementary file 5 — Additional file 5: Figure S2. Sciatic nerve functional index (SFI) and tibial functionality index (TFI) during 12 weeks in the Sham, autograft, PCL, and PCL+ rMSC groups. (a) SFI, (b) TFI. The line red represents the values of the PCL + MSC group. The values were obtained weekly and are represented as mean ± SEM. p < 0.05*; p < 0.01**; p < 0.001***. [file 13287_2021_2315_MOESM5_ESM.tiff]

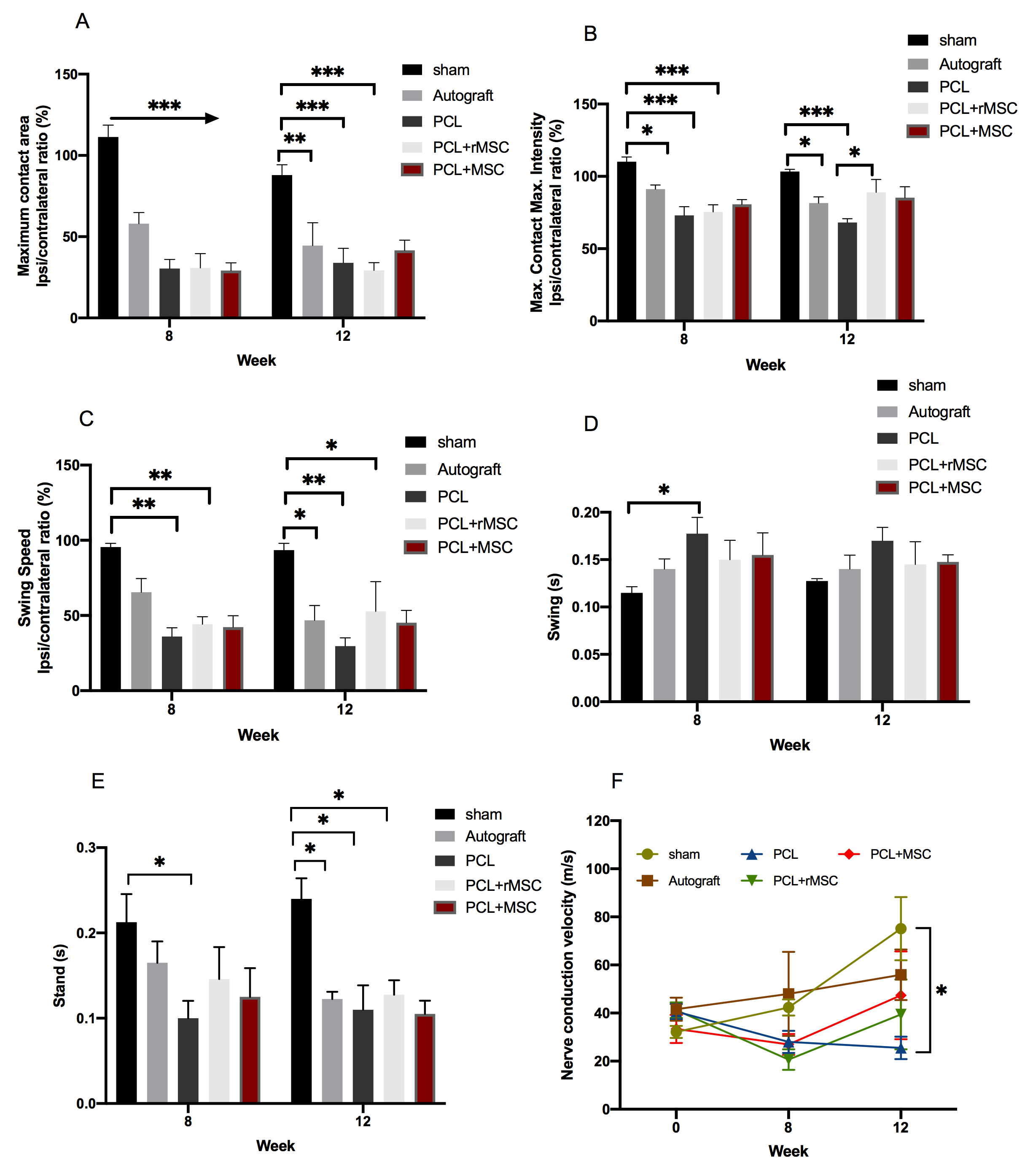

Supplement: Supplementary file 6 — Additional file 6: Figure S3. Gait analysis using the CatWalk platform and nerve conduction velocity (NCV) at 8 and 12 weeks, comparing the Sham, autograft, PCL, and PCL + rMSC groups. (a) Maximum contact area, (b) maximum contact intensity, (c) swing speed, (d) swing, (e) stand time, and (f) NCV (m/s). The red bar and line characterize the PCL + MSC group. The values obtained are represented as mean ± SEM. p < 0.05*; p < 0.01**; p < 0.001***. [file 13287_2021_2315_MOESM6_ESM.tiff]

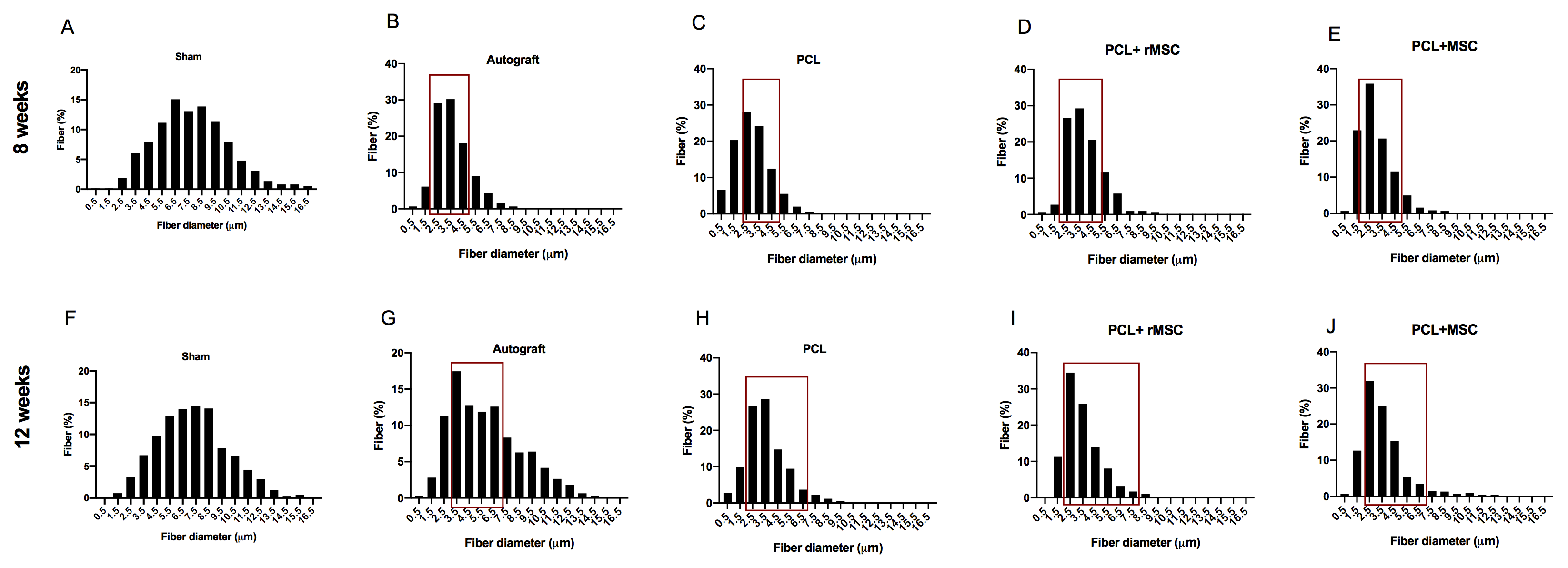

Supplement: Supplementary file 7 — Additional file 7: Figure S4. Frequency distribution of myelinated fiber diameter at 8 (a-e) and 12 weeks (f-j) after the lesion in the sham, autograft, PCL, PCL + rMSC and PCL + MSC groups. Similar values in the frequency distribution of myelinated fiber diameter were observed among PCL + rMSC and PCL + MSC groups at 8 and 12 weeks. The red boxes highlight frequency intervals with better percentages among autograft, PCL and PCL + MSC groups. [file 13287_2021_2315_MOESM7_ESM.tiff]

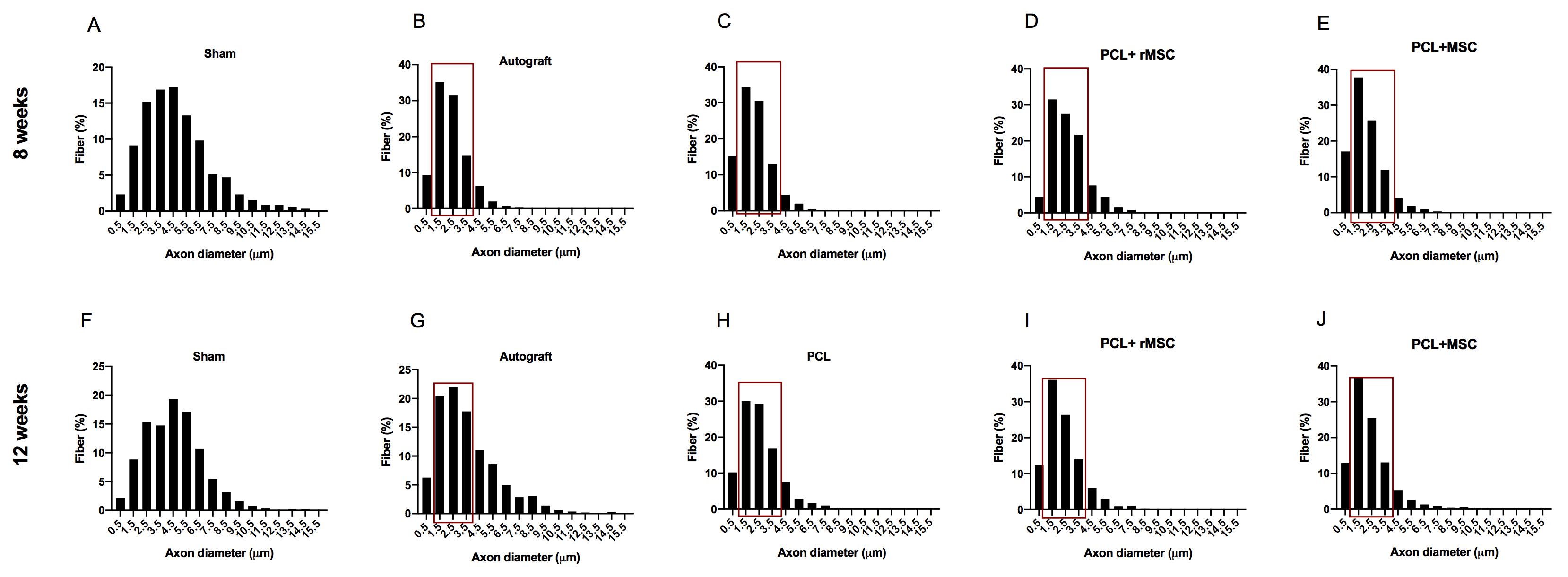

Supplement: Supplementary file 8 — Additional file 8: Figure S5. Frequency distribution of axon diameter at 8 (a-e) and 12 weeks (f-j) after the lesion in the sham, autograft, PCL + rMSC and PCL + MSC groups. Similar values in the frequency distribution of axon diameter were observed among PCL + rMSC and PCL + MSC groups at 8 and 12 weeks. Red boxes highlight frequency intervals with better percentages among autograft, PCL and PCL + MSC groups. [file 13287_2021_2315_MOESM8_ESM.tiff]

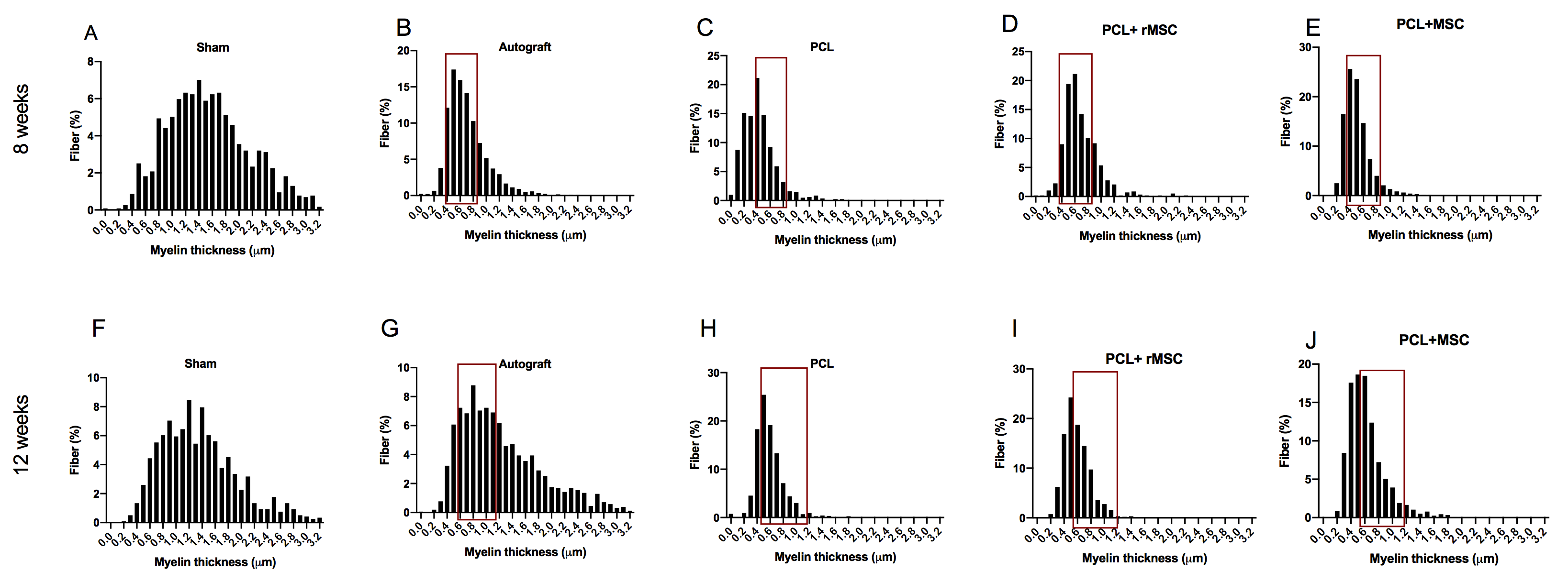

Supplement: Supplementary file 9 — Additional file 9: Figure S6. Frequency distribution of the myelin thickness in the Sham, autograft, PCL, PCL + rMSC and PCL + MSC groups at 8 weeks (a-e) and 12 weeks (f-j). At 12 weeks, myelin thickness was superior in the PCL + MSC group compared with the PCL and PCL + rMSC groups. Red boxes highlight frequency intervals with better percentages among the groups. [file 13287_2021_2315_MOESM9_ESM.tiff]

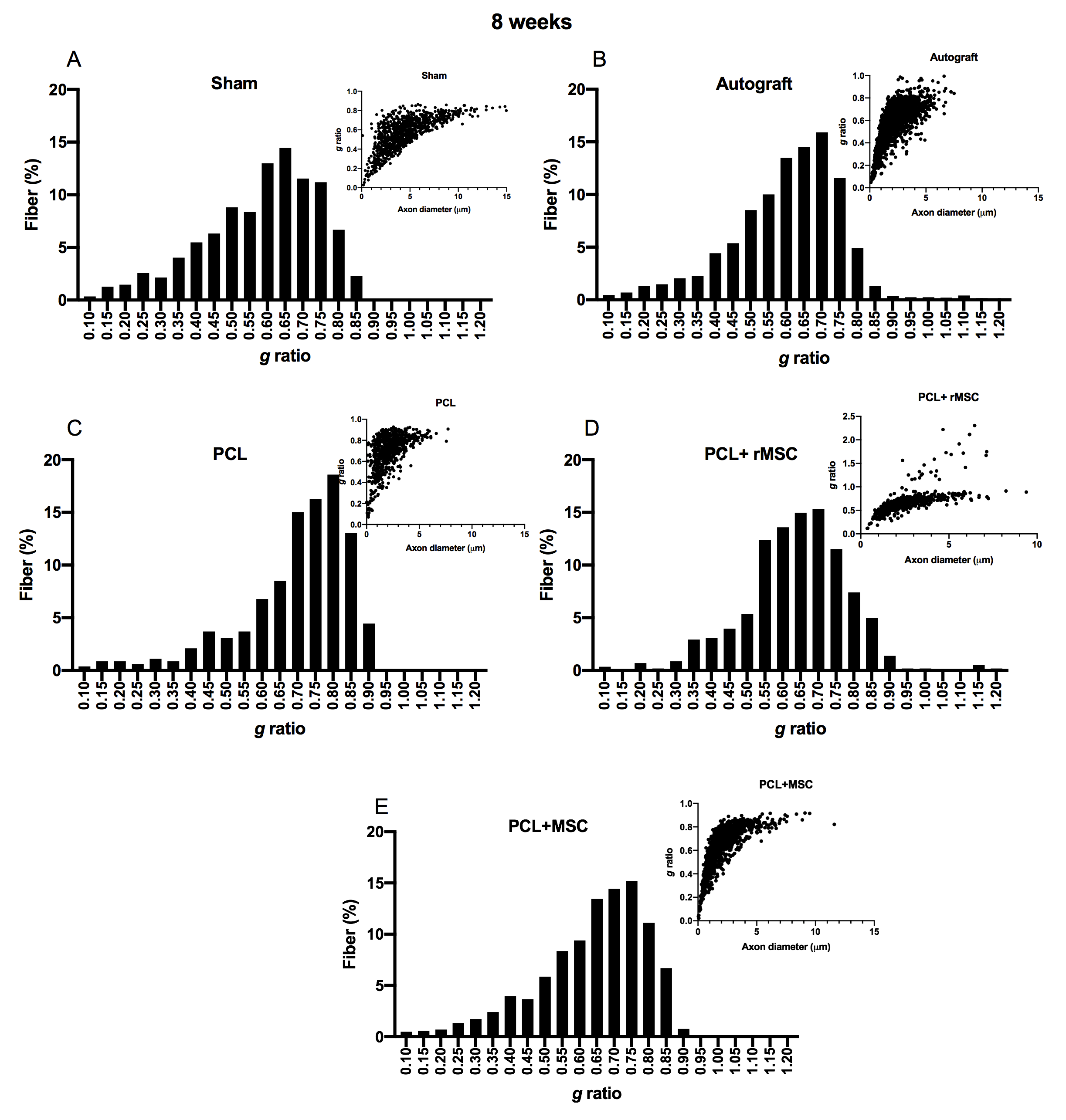

Supplement: Supplementary file 10 — Additional file 10: Figure S7. Frequency distribution of “g” ratio and dot plot of “g” ratio/axon diameter thickness in the Sham, autograft, PCL, PCL + rMSC and PCL + MSC groups at 8 weeks (a-e). Note the shift towards an increase in the diameter of the myelinated axon in the autograft and PCL + MSC groups when compared to the PCL and PCL + rMSC groups at 8 weeks. [file 13287_2021_2315_MOESM10_ESM.tiff]

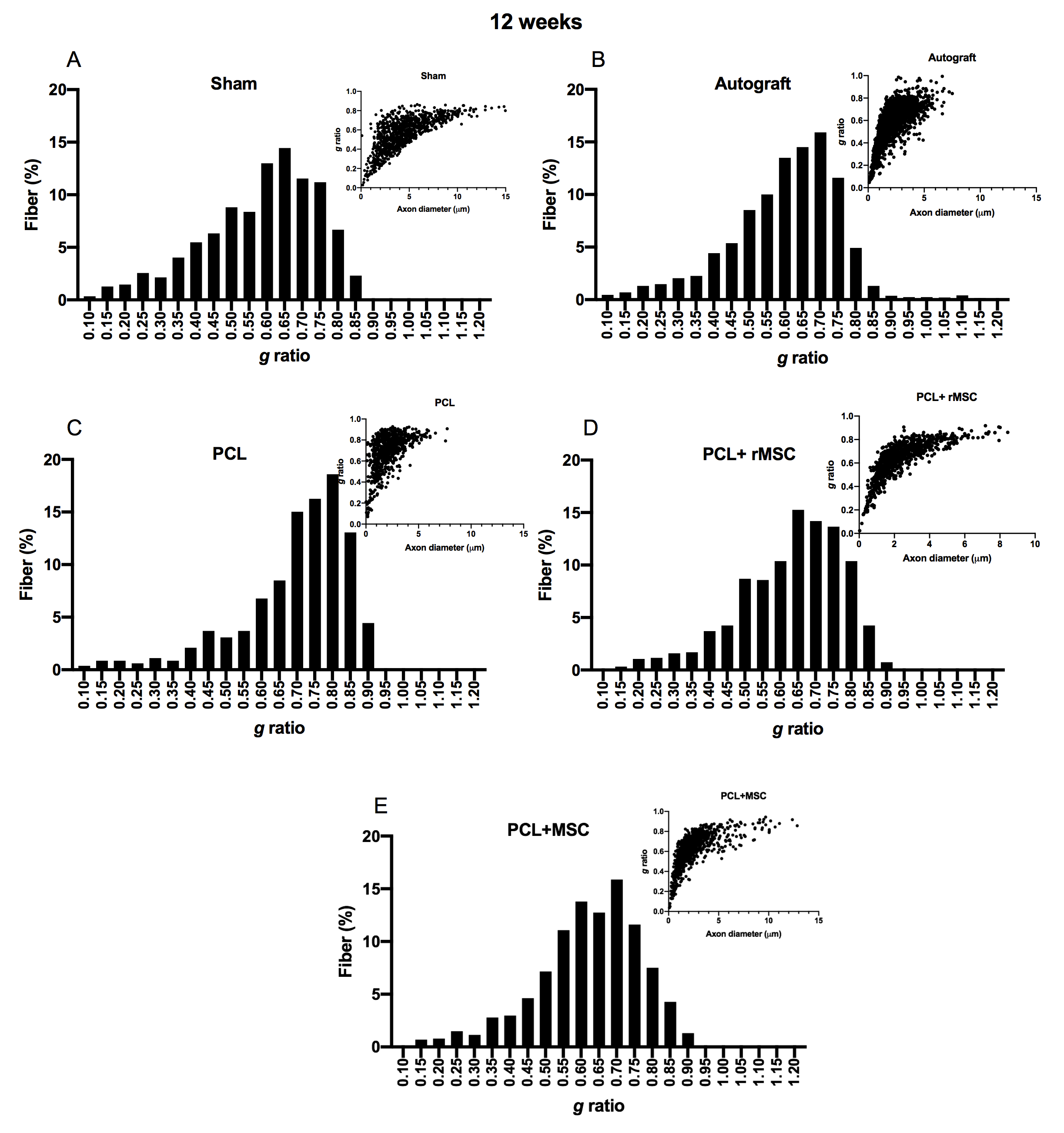

Supplement: Supplementary file 11 — Additional file 11: Figure S8. Frequency distribution of “g” ratio and dot plot of “g” ratio/axon diameter thickness in the Sham, autograft, PCL, PCL + rMSC and PCL + MSC groups at 12 weeks (a-e). At 12 weeks, frequency distribution was similar in PCL + MSC and PCL + rMSC groups. [file 13287_2021_2315_MOESM11_ESM.tiff]
